# Non-Canonical Amino Acids in Analyses of Protease Structure and Function

**DOI:** 10.3390/ijms241814035

**Published:** 2023-09-13

**Authors:** Peter Goettig, Nikolaj G. Koch, Nediljko Budisa

**Affiliations:** 1 Department of Pharmaceutical and Medicinal Chemistry, Institute of Pharmacy, Paracelsus Medical University, Strubergasse 21, 5020 Salzburg, Austria; 2 Biocatalysis Group, Technische Universität Berlin, 10623 Berlin, Germany; nikolaj.koch@unibas.ch; 3 Bioanalytics Group, Institute of Biotechnology, Technische Universität Berlin, 10623 Berlin, Germany; nediljko.budisa@umanitoba.ca; 4 Department of Chemistry, University of Manitoba, Winnipeg, MB R3T 2N2, Canada

**Keywords:** bioorthogonal tags, cross-linking, click chemistry, genetic code expansion, non-canonical amino acids, inhibitory potency, pharmaceutical compound, protease specificity, proteinogenic amino acid, unnatural amino acid

## Abstract

All known organisms encode 20 canonical amino acids by base triplets in the genetic code. The cellular translational machinery produces proteins consisting mainly of these amino acids. Several hundred natural amino acids serve important functions in metabolism, as scaffold molecules, and in signal transduction. New side chains are generated mainly by post-translational modifications, while others have altered backbones, such as the β- or γ-amino acids, or they undergo stereochemical inversion, e.g., in the case of D-amino acids. In addition, the number of non-canonical amino acids has further increased by chemical syntheses. Since many of these non-canonical amino acids confer resistance to proteolytic degradation, they are potential protease inhibitors and tools for specificity profiling studies in substrate optimization and enzyme inhibition. Other applications include in vitro and in vivo studies of enzyme kinetics, molecular interactions and bioimaging, to name a few. Amino acids with bio-orthogonal labels are particularly attractive, enabling various cross-link and click reactions for structure-functional studies. Here, we cover the latest developments in protease research with non-canonical amino acids, which opens up a great potential, e.g., for novel prodrugs activated by proteases or for other pharmaceutical compounds, some of which have already reached the clinical trial stage.

## 1. Introduction

**Figure 1:**
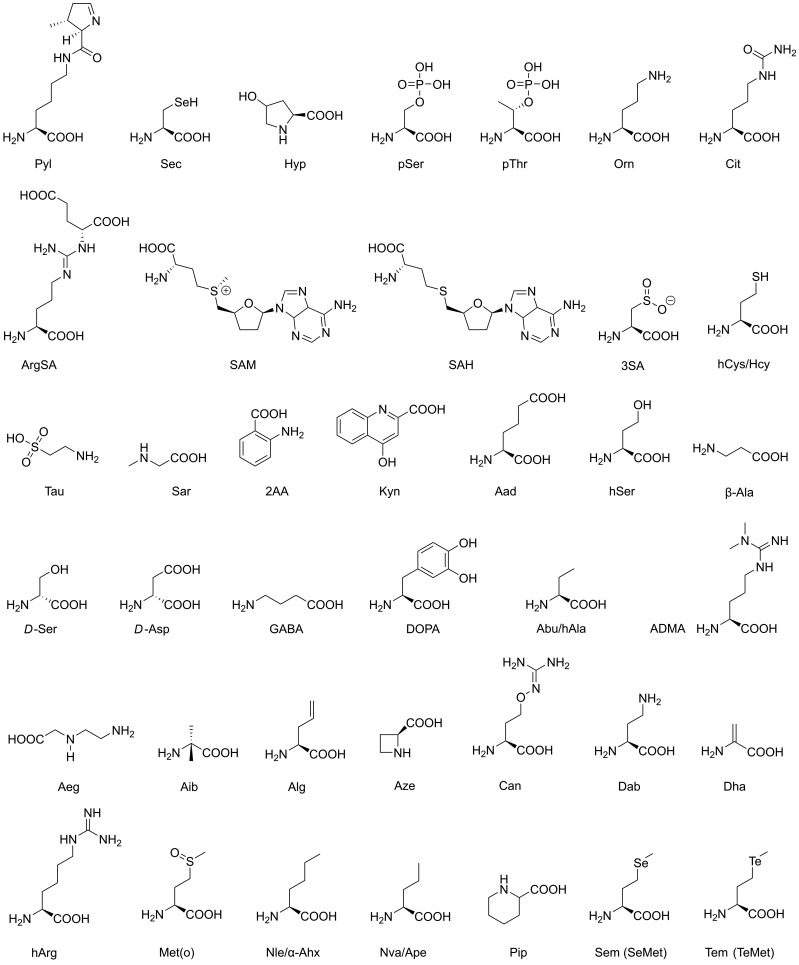
Natural non-canonical amino acids. More details on these molecules, such as occurrence, biological functions, and usage, are given in Table 1. Details on nomenclature (IUPAC) and CAS numbers (Chemical Abstract Services) are given in Appendix A.

All natural proteins contain the 20 canonical or standard L-amino acids (cAAs) present in all living organisms and viruses. In addition, archaea, bacteria, and eukaryotes possess selenocysteine (Sec) as the 21st proteinogenic amino acid [1]. Another proteinogenic amino acid is pyrrolysine (Pyl), which is present in some archaea and bacteria (Figure 1) [2,3]. Remarkably, Pyl is genetically encoded and read from UAG stop codons (*amber*) in frame. A natural orthogonal pair of aminoacyl-tRNA synthetase (PylRS) and tRNA^Pyl^ enables the insertion of Pyl into proteins. Similarly, in-frame reading of UGA stop codons (*opal*) allows to incorporate Sec into proteins. However, the mechanism is more complicated, as seryl-tRNA synthetase generates seryl-tRNA^Sec^, followed by conversion to selenocysteyl-tRNA^Sec^. Moreover, Sec incorporation requires a specific elongation factor at the ribosome and recognition elements in the mRNA [4]. The molecular properties of many proteinogenic amino acids are often altered by post-translational modifications (PTM), such as disulfide formation, glycosylation, phosphorylation, hydroxylation, methylation, lipidation or decarboxylation, to name the most important ones. About 500,000 PTM sites have been catalogued in mammalian proteomes alone, while archaea require many PTMs to survive in extreme environments, and bacteria share some PTMs with eukaryotes [5,6,7].

However, the range of natural amino acids from biological sources is much larger than the 20 canonical or 22 proteinogenic ones, as up to 900 non-canonical amino acids (ncAAs) are known to date. However, there is good evidence that some amino acids are of extraterrestial abiotic origin. For example, after the fall of the Murchison meteorite in Australia in 1969, it was soon found to contain Gly, Ala, Val, Pro, Glu and ncAAs, such as 2-methylalanine and sarcosine [8]. More than 100 of these primordial amino acids have been discovered in meteorites to date, which is not surprising, since the intergalactic space is abundant in all necessary precursor molecules. Most chiral amino acids from meteorites occur as racemic mixtures, while some samples exhibited an excess of the L-form or, less frequently, of the D-form [9]. Recently, a new family of hydroxyamino acids unknown on Earth, e.g., β-aminomethyl succinic acid, was discovered in the Murchison meteorite [10].

In living cells, some non-proteinogenic amino acids are essential in the metabolism, e.g., ornithine (Orn), citrulline (Cit) and argininosuccinate (ArgSA) in the urea cycle [11]. Others function as neuronal messengers similar to GABA, such as β-amino acids and D-amino acids, which are derived from canonical amino acids, since corresponding racemases exist in mammalian tissues (Figure 1) [12]. *D*-serine (*D*-Ser) acts as co-agonist of the N-methyl-*D*-aspartate glutamate or NMDA receptors, whereas *D*-Asp is a major regulator of adult neurogenesis and a crucial component in the development of endocrine function [13]. However, *D*-Asp is increasingly found during aging in long-lived proteins, since Lα-Asp spontaneously racemizes via a succinimidyl intermediate to Lβ-, Dα- and Dβ-isomers [14].

β-amino acids are also present in biomolecules, e.g., as a building block of the natural anticancer drug taxol (paclitaxel) or, like β-Ala, as a component of vitamin B5 (pantothenate), which is a precursor of coenzyme A [15,16]. In addition, natural ncAAs, such as dimethylarginine (ADMA) Alg and homoarginine (hArg), participate in the physiological nitric oxide processes, involving atherosclerosis and atherogenesis. In nature, there are several other “homo-amino acids”, such as homoserine, homocysteine (hCys) or homophenylalanine (hPhe), which are one methylene group (CH_2_) longer than their canonical counterparts (Figure 1) [17,18]. S-adenosylmethionine (SAM), S-adenosyl homocysteine (SAH), hCys, 3-sulfinoalanine (3SA) and taurine (Tau) participate in the sulfur metabolism and the transfer of methyl groups, whereby hCys and Tau play a role as neurotransmitters [19,20]. Cyanobacteria produce some ncAAs, such as β-methylamino-*L*-alanine (BMAA), 2,4-diaminobutyric acid (Dab) and N-2-aminoethyl-glycine (Aeg), which have neurodegenerative potential for humans [21]. In the following, L-amino acids are not explicitly designated, in contrast to D-amino acids (Figure 1, Table 1; for details of nomenclature, see Appendix A).

**Figure 2:**
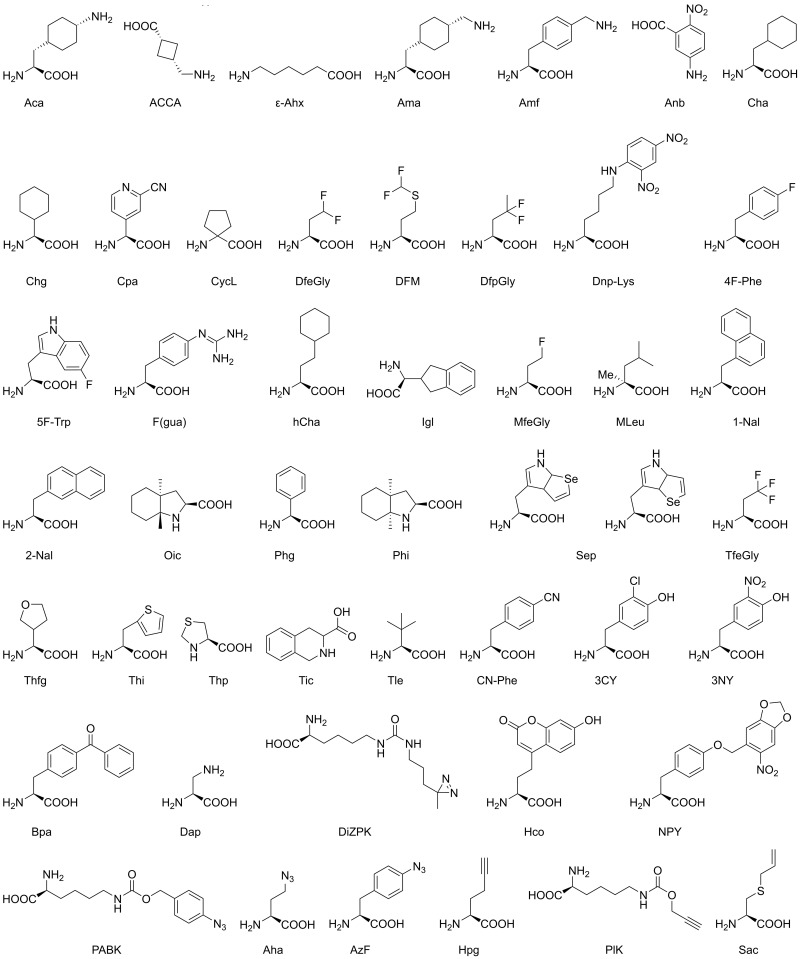
Unnatural non-canonical amino acids. Further details on these molecules, such as experimental applications, are given in Table 2. Details on nomenclature (IUPAC) and CAS numbers (Chemical Abstract Services) are given in Appendix B.

Regarding the terminology, a clear distinction can be made between proteinogenic, non-proteinogenic, natural non-canonical (ncAA) and non-natural amino acids (nnAA), which corresponds to the more frequently used term unnatural amino acids (uAA). Unnatural or synthetic amino acids are often designated ncAAs as well (Figure 2, Table 2, nomenclature details in Appendix B). Nevertheless, it is necessary to use these terms separately, to retrieve the relevant publications in PUBMED. Accordingly, the appropriate terminology is used in this review to distinguish these amino acids, preferentially using ncAA for natural ones and uAA for synthetic ones, which are often referred to as ncAAs or, more rarely, as “unusual amino acids” in the literature as well. With respect to methods, and in general considerations, the term ncAA comprises natural and unnatural amino acids. Protease designations, such as A01.001 for the aspartic protease pepsin, follow the nomenclature of the MEROPS database (https://www.ebi.ac.uk/merops/, accessed on 19 August 2023) [22]. Associated with MEROPS, and published about a decade ago, the most comprehensive and systematic work on proteases is the *Handbook of Proteolytic Enzymes*, with descriptions of proteolytic mechanisms [23,24]. Our main goal is to present the highly significant and widely applied usage of ncAAs in basic and commercial research of proteases. Thus, we provide a comprehensive overview, spanning the earliest beginnings to the most recent developments, with respect to methods and numerous examples of individual proteases.

**Table 1:** Natural non-canonical amino acids, occurrence and functions.

AA	Standard Name	Type and Occurrence	Functions and Usage
Sec	selenocysteine	all organisms (ao)	redox processes
Pyl	pyrrolysine	archaea and bacteria	methyltransferase catalysis
Hyp	hydroxyproline	vertebrates (ve)	PTM, collagen stability
pSer	phosphoserine	eukaryotes (eu)	PTM, signaling/cancer
pThr	phosphothreonine	eu	PTM, signaling/cancer
Orn	ornithine	ao	urea cycle
Cit	citrulline	ao	urea cycle
ArgSA	argininosuccinic acid	ao	urea cycle
SAM	S-adenosylmethionine	ao	DNA methylation (eukaryotes)
SAH	S-adenosylhomocysteine	ao	sulfur metabolism
3SA	3-sulfinoalanine	ao	sulfur metabolism
hCys/Hcy	homocysteine	ao	sulfur metabolism
Tau	taurine	ao	sulfur metabolism
Sar	sarcosine	ao	glycine biosynthesis
2AA	anthranilic acid	ao	tryptophan biosynthesis
Kyn	kynurenic acid	ao	tryptophan degradation
Aad	aminoadipic acid	ao	lysine biosynthesis
hSer	homoserine	ao	methionine metabolism
β-Ala	β-alanine	eu	vitamin B5 component
*D*-Ser	*D*-serine	ve	neurotransmitter, coagonist NMDA receptor
*D*-Asp	*D*-aspartic acid	ve	neurotransmitter, agonist NMDA receptors
GABA	γ-aminobutyric acid	ve	neurotransmitter, GABA receptors
DOPA	3-hydroxytyrosine	ve	neurotransmitter, dopamine precursor
Abu/hAla	α-aminobutyric acid	ve	metabolite
ADMA	dimethylarginine	ve	nitric oxide synthase regulator
Aeg	N-2-aminoethylglycine	cyanobacteria	toxin
Aib	α-aminoisobutyric acid	ao	metabolite
Alg	allylglycine		rare metabolite
Aze	azetidine-2-carboxylic acid	plants, *Convallaria majalis*	toxin
Can	canavanine	plants, *Leguminosae*	toxin
Dab	2,4-diaminobutyric acid	cyanobacteria	toxin
Dha	dehydroalanine	bacteria, *Lactococcus lactis*	lantibiotics
hArg	homoarginine	ao	bacterial growth inhibitor
Met(o)	methionine sulfoxide	ao	aging proteins
Nle/α-Ahx	norleucine	bacteria	
Nva/Ape	norvaline	bacteria	
Pip	pipecolic acid	ao	immunity regulator
Sem/SeMet	selenomethionine	Met analog in proteins	X-ray crystallography
Tem/TeMet	telluromethionine	Met analog in proteins	X-ray crystallography

**Table 2:** Unnatural non-canonical amino acids, usage, and function.

AA	Standard Name	Characteristics and Usage
Aca	*trans*-4-aminocyclohexylalanine	substrate, inhibitor
ACCA	*cis*-3-aminomethylcyclobutane carboxylic acid	inhibitor
ε-Ahx	aminocaproic acid/6-aminohexaonic acid	substrate, inhibitor, ABP
Ama	*trans*-4-aminomethylcyclohexylalanine	substrate, inhibitor
Amf	4-aminomethylphenylalanine	substrate, inhibitor
Anb	5-amino-2-nitrobenzoic acid	substrate, inhibitor, ABP
Cha	cyclohexylalanine	substrate, inhibitor
Chg	cyclohexylglycine	inhibitor
Cpa	3-(2-cyano-4-pyridyl)alanine	inhibitor
CycL	cyclo-leucine/cyclo-Leu	inhibitor
DfeGly	difluoroethylgycine	inhibitor, ABP
DFM	difluoromethionine	protein modification
DfpGly	difluoropropylgycine	inhibitor
Dnp-Lys	N(6)-(2,4-dinitrophenyl)lysine	substrate (fluorophore)
4F-Phe	4-fluorophenylalanine (^19^F)	NMR
5F-Trp	5-fluorotryptophan (^19^F)	NMR
F(gua)	4-guanidinophenylalanine	substrate, inhibitor, ABP
hCha	homocyclohexylalanine	substrate, inhibitor
Igl	2-indanylglycine	substrate, ABP
MfeGly	monofluoroethylgycine	inhibitor
MLeu	α-methyl-leucine	inhibitor
1-Nal	1-naphthylalanine	inhibitor
2-Nal	2-naphthylalanine	inhibitor
Oic	octahydroindole-2-carboxylic acid	substrate, inhibitor, ABP
Phg	phenylglycine	substrate, inhibitor
Phi	perhydroindol-2-carboxylic acid	inhibitor
Sep	selenotryptophans (selenolo[3,2-b]pyrrole/[2,3-b]pyrrole)	X-ray cystallography
TfeGly	trifluoroethylgycine	inhibitor, ABP
Thfg	tetrahydrofuranylglycine	inhibitor
Thi	3-(2-thienyl)alanine/β-thienylalanine or 3-(3-thienyl)alanine	substrate, inhibitor
Thp	thioproline	inhibitor
Tic	1,2,3,4-tetrahydroisoquinoline-3-carboxylic acid	substrate, inhibitor
Tle	*tert*-leucine/*tert*-butyl-glycine	substrate, inhibitor, ABP
CN-Phe	4-cyanophenylalanine	fluorophore
3CY	3-chlorotyrosine	protein modification
3NY	3-nitrotyrosine	protein modification, fluorophore
Bpa	4-benzoylphenylalanine	photo-crosslinker
Dap	2,3-diaminopropionic acid	ABP for Ser/Cys proteases
DiZPK	3-(3-methyl-3H-diazirine-3-yl)-propamino(carbonyl-Nε-lysine)	protein modification/photo-crosslinker
Hco	7-(hydroxy-coumarin-4-yl)-ethylglycine	protein modification/fluorophore
NPY	nitropiperonyltyrosine	caging and decaging
PABK	Nε-4-azidobenzyloxycarbonyllysine	click reactant, caging and decaging
Aha	azidohomoalanine	click reaction, 1,3-dipolar cycloaddition
AzF	4-azidophenylalanine	1,3-dipolar cycloaddition
Hpg	homopropargylglycine	1,3-dipolar cycloaddition
Plk	N-propargyllysine	1,3-dipolar cycloaddition
Sac	S-allylcysteine	photo-click reaction

## 2. Methods for the Incorporation of ncAAs in Peptides and Proteins

Non-canonical amino acids (ncAAs) can be introduced into peptides and recombinant proteins using various in vivo and in vitro methods or by specific chemical and enzymatic modifications. As these methods are very numerous, we recommend interested readers to study the referenced literature of this review to learn all the necessary details. These insertions or modifications can be made to the protein backbone or on the side chains of amino acids, providing useful alterations to the properties of the protein. In nature, alterations of the backbone of proteins usually occur after the translation process through post-translational modifications. On the other hand, when we want to introduce changes to protein backbones that are not naturally occurring, we can use proline analogs to modify them in a living organism [25]. In vitro ribosomal translation systems can be used to introduce other modifications, such as D-, β- or γ-amino acids, into target polypeptides. Additionally, specific engineered systems can incorporate α-hydroxy acids into proteins in response to in-frame *amber* codons [26]. Overall, the incorporation of ncAAs into proteins provides a powerful tool for protein engineering, enzyme catalysis, biomedicine and biotechnology [27].

### 2.1. Chemical Modification of Standard Amino Acids

Historically, chemical modification of natural amino acid residues was the first method to introduce unnatural side chains into peptides and proteins for analytical and preparative applications, as Cys alkylation of the enzyme glyoxalase with iodoacetamide dates back to 1933 [28,29]. Proteases can be repurposed as ligases to synthesise peptides with unnatural side chains. For example, enzymatic synthesis of corresponding peptides is a different route using proteases as ligases, like the cysteine protease papain for the generation of the tripeptide ethyl ester Ala-Aib-Ala-OEt containing α-aminoisobutyric acid [30]. Various proteases have been widely used as ligases to synthesize polypeptides and protein, especially with ncAAs [31].

Site-specific chemical modifications of residues involved in catalysis were used in several functional investigations of proteases. Mutation of the catalytic Lys145 to Cys rendered the *Escherichia coli* signal peptidase inactive, whereas the reaction of this Cys with 2-bromoethylamine and 3-bromopropylamine restored the activity of the resulting γ-thia-lysine and its homolog [32]. Ser-to-Cys mutations of subtilisin from *Bacillus lentus* were used to explore the S2, S1 and S1′ pocket specificity via chemical modification to Cys-SR, with a variable length of R and increasing charges [33]. Several investigated variants remained active, but positive and negative charges, as well as a branched side chain at position 222, reduced the catalytic efficiency 100-fold. Essentially, chemical modification of Cys62 (S2) to methanethiosulfonate, similar to Cys156/166 (S1) and Cys217 (S1′), reduced the catalytic efficiency stepwise down to 10% of the wild-type enzyme by increasing the negative charge in each position [34,35].

### 2.2. Substitution of Specific cAAs by ncAAs in Auxotrophy-Based Methods and Its Relevance for Structural Biology

As early as 1957, in a ground-brea_ki_ng experiment with the Met-auxotrophic *E. coli* ML304d strain, Cowie and Cowen showed that basically all methionine residues in newly synthesized proteins could be replaced by selenomethionine (SeMet) [36]. In 1962, already about 30 ncAAs, such as norleucine (Nle, α-Ahx), fluoro-Phe, Aza-Trp and methyl-Trp, were incorporated into proteins of corresponding auxotrophic bacteria [37]. Typically, the method worked only for Met, Leu, Ile, Phe, Tyr, Trp, Arg and Lys analogs. Since the early 1990s, ncAAs have been incorporated into target proteins by expression in *E. coli*, mostly for structural studies with X-ray crystallography with multi-wavelength anomalous dispersion (MAD) [38,39]. Auxotrophic bacteria were employed, which cannot synthesize a particular amino acid, such as methionine. The medium was then supplemented with 19 standard amino acids and selenomethionine (SeMet, Sem) or, in rare cases, telluro-methionine (TeMet, Tem, Figure 1) [40,41]. The method was extended, e.g., by incorporating selenium containing Trp (Sep), which exhibited an anomalous signal of the selenium sites in a crystallized model protein (Figure 2) [42].

**Figure 3:**
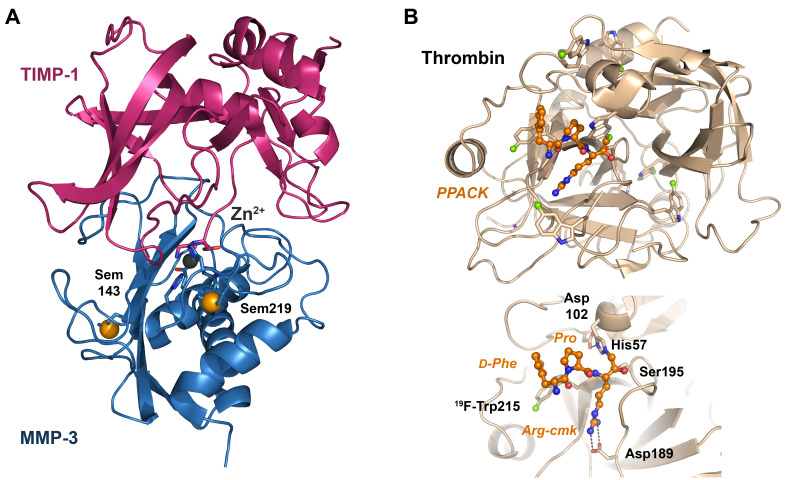
Non-canonical amino acids in structure-function studies. (**A**) Human MMP-3 in complex with its natural inhibitor TIMP-1. Two selenomethionine residues (Sem, with orange spheres) were sufficient to determine the structure by MAD phasing at 2.8 Å resolution (PDB code 1UEA). The catalytic Zn^2+^ (black sphere) is coordinated by three His, a Glu and the N-terminus of TIMP-1. (**B**) Thrombin in which all Trp residues were replaced by 5-^19^F-Trp via the auxotrophy-based reassigned sense codon method (PDB 6V64). ^19^F-Trp allowed to measure molecular dynamic processes by NMR, with the fluorine atoms depicted as green spheres. The covalently bound chloromethyl ketone inhibitor PPACK, shown as a ball-and-stick model, contains the modified Arg-cmk, occupying the S1 pocket, a Pro in the S2 pocket and a *D*-Phe binding to the S4 pocket formed largely by F-5-Trp215. The residues of the catalytic triad His57, Asp102 and Ser195 are shown as sticks, as is Asp189 at the bottom of the S1 pocket, which confers the specificity for basic substrates.

Since about 1985, undulator beamlines have been available at synchrotrons, which allowed to adjust distinct X-ray wavelengths for anomalous scattering of transition and heavy metals, to obtain phase information for structure determination [43]. In the following years, anomalously scattering atoms, either soaked in protein crystals or incorporated in proteins themselves, were utilized to collect X-ray diffraction data sets with phase information [44]. Crystals of the natural ncAA selenolanthionine allowed to determine the anomalous scattering factors f′ and f″ of selenium at the K edge with polarized synchrotron radiation [45,46]. Moreover, detectors were improved, in particular charged coupled devices (CCD) [47]. A complex of selenobiotin and streptavidin was the basis for structure determination with the multi-wavelength anomalous diffraction method (MAD) [48]. Already in 1989, Tem was incorporated into fungal proteins, and the first incorporation of Tem in *E. coli* dihydrofolate reductase was reported in 1994 [49,50]. Incorporation of Sem to 100% and Tem to 75% was achieved for *Staphylococcus aureus* pyrrolidone carboxyl peptidase, with unchanged enzymatic properties [40]. The application of the method of reassigned sense codons for auxotrophic *E. coli* allowed to incorporate methionine analogs, such as Sem and Tem, at high levels [41]. Subsequently, Tem incorporation in model proteins facilitated their structure determination both with the MAD and with the multiple isomorphous replacement (MIR) approach [51]. An excellent overview of biological research with selenium and tellurium analogs of Met and Cys, such as Sem, Tem, Sec and Tec (TeCys), was provided by Musiol and Moroder [52]. Based on the incorporation of Sem, the complex of MMP3/TIMP1 is a fine example of structure determination with the MAD method (Figure 3A) [53].

Meanwhile, single-wavelength anomalous dispersion (SAD) became the dominant method for de novo protein structure determination; since the year 2007, more deposited PDB coordinates were obtained by SAD than by MAD [54,55]. In addition to this development, the quantitative incorporation of Sem into proteins expressed in yeast, insect and mammalian cells was established for structural biology [56,57,58]. Although many atoms can be utilized, even sulfur, phosphorus and other light atoms, Sem-SAD is still the most common approach. Also, the extremely intense radiation of X-ray free-electron lasers (XFEL) is suitable for SAD phasing with protein microcrystals [59]. This method is constantly being improved, e.g., with the SAD-SMAR algorithm, which facilitates to determine the anomalous scattering substructure in a highly efficient manner [60]. Currently, about 30% of all crystal structures are solved by experimental phasing methods; the rest only requires molecular replacement with a related protein model [61].

As the genetic information of an organism can be encoded with only 30–40 sense codons, there is potential to recode more than 20 sense codons with ncAAs [62]. Tirrell and co-workers have proposed an alternative approach to stop codon suppression (SCS) by incorporating ncAAs at rare sense codons [63]. In 2014, Budisa and Bohlke attempted to exploit the degeneracy of the genetic code by freeing rare sense codons from their original coding roles and reprogramming them to code for specific ncAAs permanently [64]. However, with methods for reassigning sense codons still in their infancy, the field is currently dominated by stop codon suppression technologies, which have been increasingly improved and expanded [65,66,67]. In an unusual approach of the reassigned sense codon, cell-free protein synthesis with an *E. coli* 30S ribosomal extract was applied, in order to replace all natural Asp and Phe positions with 2-naphthylalanine (2-Nal) or 4-phenylazophenylalanine in the HIV protease [68]. To this end, the tRNAs for Asp and Phe had to be inactivated by corresponding antisense mRNA, resulting in an acceptable translation efficiency.

Special cases of modified cAAs and ncAAs are isotope-labeled ones, which are widely employed in nuclear magnetic resonance spectroscopy (NMR spectroscopy) and mass spectrometry (MS). Hereby, hydrogen, carbon, nitrogen and many other atom nuclei are replaced by deuterium (^2^H/D), ^13^C, ^15^N, ^19^F, ^31^P, etc. to solve structures of small and large biomolecules, investigate their dynamics, diversify structures and discover new drugs [69,70,71,72]. In an exemplary study, the reassigned sense codon method was utilized to incorporate 5-^19^F-Trp (an isotope-labeled 5F-Trp) into thrombin, to measure the dynamics of the protease by NMR, as well as enzyme kinetics, which was further supported by X-ray crystallography (Figure 3B) [73]. In addition, NMR with labeled AAs is useful in solving membrane protein structures, e.g., in the solid state, as well as in the emerging field of metabolomics [74,75]. Isotope labeling of proteins can be utilized in combination with NMR and MS or in MS alone, as for proteomics [76,77]. Numerous overview articles describe other promising applications of auxotrophy-based methods [78,79].

### 2.3. Site-Directed Insertion of ncAAs Using Orthogonal Pairs

In order to incorporate a non-canonical amino acid (ncAA) into a protein sequence, a distinct set of aminoacyl-tRNA synthetase (aaRS) and tRNA molecules is needed. These molecules must be orthogonal, meaning they must not interact with the endogenous aaRS-tRNA pairs, and should be capable of reading stop codons in the target mRNA sequence to allow the accurate incorporation of the desired ncAA [80]. Typically, the selection of molecular machineries capable of incorporating ncAAs is performed by generating gene libraries of the incorporating synthetase [81]. Two Archaean systems, the *Methanocaldococcus jannaschii* tyrosine pair (MjTyrRS:tRNA^Tyr^) and the *Methanosarcina* pyrrolysine pair (PylRS:tRNA^Pyl^), have been used in *Escherichia coli* [82,83]. These systems enable the site-specific incorporation of diverse ncAAs into proteins in vivo, providing a powerful tool for creating novel proteins with unique properties [84]. In the contemporary literature, there are many excellent reviews that cover these topics in detail [85]. Therefore, interested readers are encouraged to consult these reviews for further information [86,87].

Over the past two decades, our research group and others have discovered that pyrrolysyl-tRNA synthetase (PylRS) is an especially versatile enzyme that can incorporate diverse amino acid substrates into proteins. As already mentioned, the natural substrate of PylRS is pyrrolysine (Pyl), a rare proteinogenic amino acid found in few proteomes, mainly in methanogenic microorganisms (Figure 1, Table 1) [88,89,90]. Pyl is a lysine analog with a 4-methyl-pyrroline-5-carboxylate ring attached to its side chain. Pyl is recognized and charged onto tRNA^Pyl^ by PylRS, which is a class II aminoacyl-tRNA synthetase, with the cognate tRNA anticodon CUA, creating a natural PylRS:tRNA^Pyl^ orthogonal pair that fulfills all requirements for a natural orthogonal translation system (OTS) [91]. This OTS is capable of in-frame stop codon suppression and subsequent site-specific incorporation of an ncAA into a recombinant target protein. Overall, PylRS provides a powerful tool for the incorporation of ncAAs into proteins, enabling the creation of novel proteins with unique properties [92].

Expanding the genetic code with orthogonal pairs has enabled the site-specific incorporation of numerous ncAAs into proteins, offering great potential for mimicking post-translational modifications (PTMs) in a precisely controlled manner [93]. In contrast, traditional methods of protein modification often result in non-specific labeling, leading to a complex and heterogeneous protein mixture. This micro-heterogeneity is a critical issue when aiming to achieve specific PTMs with desired chemical modifications, both natural and unnatural. Therefore, there is a need for selective methods that enable the absolute control of the position of the reactive handle within a protein [94]. Over the past few years, it has been possible to incorporate various ncAAs into proteins in a site-specific manner using different hosts such as bacteria, yeasts, mammalian cells and even multicellular organisms like *Caenorhabditis elegans* and *Drosophila melanogaster* [95,96].

As the stop codon suppression method is very versatile and already available from biological systems, the pyrrolysine tRNA synthetase-tRNA pair is widely utilized, due to its high promiscuity regarding the acceptance of ncAAs through reassignment of the *amber* stop codon UGA [97]. To incorporate natural, post-translationally modified AAs, such as phospohorylated serine (pSer), in a site-specific manner, this method was utilized for the *amber* and the Leu codon CUA with orthogonal translation systems in *E. coli* (Figure 1) [98,99]. Apart from *E. coli*, organisms like yeast, *Leishmania* and even mammalian cell cultures were successfully employed for the incorporation of hundreds of unnatural ncAAs [92,100]. This method could be expanded by native chemical ligation, in order to incorporate specific groups in proteins for FRET measurements [101].

Engineering elongation factors and the ribosome itself is another route to generating proteins with ncAAs that has been reviewed by Cui and colleagues [102]. Based on early efforts to create orthogonal mRNA-ribosome pairs, alternative Shine–Dalgarno sequences were introduced in the mRNA and the corresponding recognition elements in the 16S rRNA [103,104]. An *E. coli* 23S rRNA with two mutations in the peptidyltransferase center allowed to incorporate *D*-Phe and *D*-Met into bacterial dihydrofolate reductase (DHFR) and insect luciferase by cell-free expression [105]. Similarly, various β-amino acids could be incorporated into DHFR, e.g., β,β-dimethyl-β-alanine [106]. Recently, it was demonstrated that whole *E. coli* cells can be labeled with fluorescent ncAAs, such as 4-cyanophenylalanine (CN-Phe) and two oxazole derivatives, which was facilitated by modified ribosomes and a PylRS-tRNA^Pyl^ orthogonal pair [107]. The novel PURE translation system produces affinity-tagged polypeptides, whereby so-called hyper-accurate ribosomes with mutations in the S12 protein significantly enhanced yields of ncAA containing peptides, exemplified for α-methyl-Cys [108]. Nevertheless, the genetic code itself can be modified by using quadruplets instead of triplets of DNA and RNA bases, as well as by unusual new base molecules [109].

## 3. Protease Substrates, Inhibitors and Activity-Based Probes with ncAAs

In general, many ncAAs confer stability to peptides, due to resistance against most proteases from the three kingdoms of life [110,111,112]. Frequently, novel ncAAs are tested as antimicrobial peptides against bacteria and fungi. For example, the peptide ILLKKLLKKI, which consisted entirely of D-amino acids, killed *Mycobacterium tuberculosis* as effectively as the non-peptidic drug rifampicin [113,114,115]. Similarly, the protease stability of the antimicrobial peptide KRLFKKLLKYLRKF was significantly increased by incorporation of *D*-Lys, *D*-Arg, 2,4-diaminobutyric acid (Dab), 2,3-diaminopropionic acid, hArg, 4-aminobutyric acid and β-2-thienylalanine (Thi) (Figure 1 and Figure 2) [116].

Numerous examples of peptidic chromogenic and fluorogenic protease substrates with ncAAs are described in the literature. Among them are natural ncAAs and synthetic uAAs, such as Nle, Orn, cyclohexylalanine (Cha) and many others, which can be most favored by a protease in distinct positions according to the Schechter–Berger nomenclature [117]. Small differences in substrate specificity of proteases are revealed by using ncAAs at certain positions of substrate analogs and activity-based probes, which allow to study different protease activities in vitro and in living cells for bioimaging [118]. So-called hybrid combinatorial substrate libraries (HyCoSuL) are suited for the investigation of various classes of proteases with cAAs and ncAAs in tetrapeptide-ACCs (7-amino-4-carbamoylmethylcoumarin) comprising P4–P1 residues [119]. Numerous activity-based probes (ABPs) have been developed to detect active proteases in vitro and in vivo. A comprehensive overview of the general topic of ABPs directed against proteases is given by Vizovišek and coworkers [120]. The first study using an ABP employed a biotinylated fluorophosphonate or FP-biotin, which was apparently able to detect several serine hydrolases in human tissues [121]. Early studies of caspases detected them in apoptotic cells with compounds like FAM–Val-Ala-Asp–fluoromethyl ketone [122]. Since some ncAA side chains bind more strongly and selectively to the specificity pockets of proteases, their systematic screening can result not only in optimal substrates and inhibitors, but also in highly specific ABPs, which facilitates the monitoring of protease activity in test tubes, body fluids or tissue samples and even in living cells. In general, ncAAs can stabilize peptides and proteins against proteolytic degradation and help in the discovery of corresponding inhibitors [123,124]. Usually, the classical and straightforward way to create small-molecule inhibitors is through synthesis with organic chemistry methods [125]. Many inhibitors are so-called peptidomimetics, which often contain non-cleavable pseudo-peptide bonds, although they are often combined with natural ncAA and uAA residues, which were already considered in early computational studies [126,127]. In the following, only a few enzyme kinetic parameters are mentioned, such as catalytic efficiency or the specificity constant (k_cat_/K_M_), preferentially inhibition constants (K_i_) instead of IC_50_ values. Equilibrium association and dissociation constants (K_a_, K_d_) are also mentioned for clarity and comparability [128,129,130].

### 3.1. Serine Proteases

#### 3.1.1. Digestive Trypsin-like Serine Proteases

The classical digestive serine proteases secreted from the human pancreas are trypsin (cationic trypsin, S01.127), chymotrypsin B (S01.152) and elastase-1 (S01.153) [131]. As the early enzymatic studies employed the bovine and porcine proteases, many experiments refer to bovine α-chymotrypsin (CTRA, S01.001), bovine trypsin (TRYB, S01.151) and porcine elastase-1 (CELA1 as well as for the human enzyme). Nevertheless, their specificity for the P1 residue in substrates remains characteristic and defines many proteases as trypsin-like (Arg/Lys), chymotrypsin-like (Phe/Tyr/Leu) and elastase-like (Ala/Val). These serine proteases and other types, such as subtilisin-like ones and α/β-hydrolases, exhibit a catalytic Ser, which is activated as a nucleophile by the two triad residues His and Asp in various structural variations [24].

Some of the work performed from the 1960s to the 1980s on chymotrypsin substrates, containing about a dozen ncAAs, was summarized in the studies of the Jakubke and Laskowski groups [132,133]. In addition, a quantitative structure–activity relationship (QSAR) was derived for the substrate fragments, i.e., essentially, the individual residues and overall log (k_cat_/K_M_) values of the substrates resulted from additive contributions of the fragments [133]. Based on these data, CTRA aldehyde inhibitors were synthesized with modified Phe residues in the P1 position, of which Tos-Phe-Ala-Thr-Phe(p-NO_2_)-CHO had a K_i_ of 11 nM [134]. A previous investigation on the chymotrypsin-like *Streptomyces griseus* protease B (SGPB, S01.262) with the uncommonly favored P1-Glu analyzed the interaction with the 55-residue-long third domain of the turkey ovomucoid inhibitor (OMTKY3, I01.003) using a homologous series of the aliphatic P1-side chains Gly, Ala, Abu, Ape (Nva), α-Ahx (Nle) and Ahp (Figure 1) [135]. In a more comprehensive work on OMTKY3, some ncAAs, such as Abu, Ape, α-Ahx and Hse, were incorporated into the P6-P3′ reactive-site loop of the inhibitor, which was assayed with CTRA and other serine proteases [132]. These studies established the sequence-to-reactivity algorithms (SRAs), that combine structural data, free energy and kinetic constants into a predictive tool [136].

Bovine pancreatic trypsin inhibitor (BPTI, aprotinin, I02.001) inhibits trypsin and chymotrypsin in a substrate-like manner, with the segment Pro13-Ile19 binding the S3–S4′ specificity sites. An enzyme kinetic and structural study of bovine β-trypsin and BPTI with the P1-Lys15 mutations to Abu, DfeGly and TfeGly demonstrated that ethyl-Gly largely reduced inhibition, whereas the fluoroethyl-Gly side chains, in particular completely fluorinated uAAs in BPTI, enhanced the inhibitory effect towards α-chymotrypsin [137].

Increasing fluorination was correlated with lower K_i_, e.g., K15Abu had a K_i_ of 1.37 µM, K15TfeGly (440 nM), MfeGly (340 nM), CHF_2_-bearing DfeGly (68 nM), DfpGly (70 nM) and PfpGly with a CF_2_-CF_3_ group (277 nM), see Figure 1 [138]. By thioester-mediated chemical ligation, an analogue of BPTI was generated, which yielded the modified disulfide Nα-ethanethiol-Gly38-Cys14 [139]. Compared to the reaction of unmodified BPTI with chymotrypsin, the equilibrium association constant K_a_ dropped from 1.7 × 10^7^ M^−1^ s^−1^ about 20-fold, which was explained by structural perturbations, as corroborated by 2D ^1^H-NMR TOCSY experiments. Using a cell-free expression system, a highly potent variant of the trypsin inhibitor ecotin with allylglycine (Alg) was obtained that allowed to cleave at the P1 reactive site upon iodine treatment and the subsequent release of active trypsin [140].

Arguably, the most comprehensibly analyzed model inhibitor is the sunflower trypsin inhibitor (SFTI-1, I12.002). A series of SFTI variants with β-/γ-amino acids and N-substituted β-Ala residues in the P1 position (residue 5) was synthesized. In addition, the N-substituted β-alanines βNhlys and βNhphe were introduced and exhibited inhibition against bovine CTRA and TRYB in vitro. The two analogs with [b3hLys5]SFTI-1 and [b3hPhe5]SFTI-1 displayed activity comparable to monocyclic SFTI-1 for TRYB and [Phe5] for CTRA with K_a_ = 1.0 × 10^10^ M^−1^ and K_a_ = 2.0 × 10^9^ M^−1^, respectively, whereby the K_i_ is about 500 pM [141]. Monocyclic linear SFTI-1 was engineered in the P1 position with Phe or N-benzylglycine (Nphe) and in the P1′ position with Hse or [N-(2-hydroxyethyl)-glycine] (Nhse), in order to efficiently inhibit CTRA, resulting in a best K_i_ of about 9 nM [142].

Spumigins are linear analogs of tetrapeptidic protease inhibitors from the cyanobacterium *Nodularia spumigena*, found in the Baltic Sea [143]. They consist of an N-terminal capping group, which is a Tyr derivative with an OH group instead of the NH_2_ group, followed by hPhe or 4-Me-hPhe, then Pro or 3-Me-Pro, and as C-terminal residue either Arg, arginal or the corresponding arginol with a CH_2_-OH moiety. It can be estimated that the variant spumigin E, which exhibits a P1 arginal, inhibits TRYB with an IC_50_ of about 200 nM. Other cyanobacteria such as *Dolichospermum planctonicum* produce a cluster of so-called radiosumins, which essentially are dipeptides comprising two unusual ncAAs with various double bonds [144]. Radiosumin C consists of two *L*-(2-amino-3-(4-amino-2-cyclohexen-1-ylidene)-propionic acid (Aayp) as P1 and P1′ residues, with two acetyl caps and a free C-terminal carboxylate, whereas radiosumin D differs slightly by a methylated 4-amino group of the P1 residue (*L*-Amyp) and a *D*-Aayp in P1′ position. Radiosumin C inhibited the three human isoforms trypsin-1 (S01.127), trypsin-2 (S01.258) and trypsin-3 (mesotrypsin, S01.174) with IC_50_ values of 1.7, >7.2 and 2.0 µM, respectively. Extracts from the marine sponge *Theonella swinhoei*, found near Madagascar, contained the three cyclotheonellazoles A-C and the related oriamide, whereby compound A inhibited CTRA with an with IC_50_ of 620 pM and CELA1 with an IC_50_ of 34 pM [145]. A capped N-terminal Gly-Ala is attached to a ring formed by amino-Ala-Nle-Ile, then an inserted carbonyl, a complex amino acid with a Tyr side chain, but a thiazole group and an ethylene group in the “backbone“, an amino acid with HO_3_S-CH_2_- as side chain that closes the ring. Another complex cyclic inhibitor of trypsin-like proteases, lyngbyastatin 4, was discovered in the marine cyanobacterium *Lyngbya confervoides*. [146]. Lyngbyastatin 4 features several ncAAs, such as N-me-Tyr, 3-amino-6-hydroxy-2-piperidone (Ahp), Abu and hTyr; it selectively inhibited chymotrypsin (IC_50_ = 300 nM) and elastase (IC_50_ = 30 nM), whereas the activity of trypsin, thrombin and plasmin was not affected.

One of the most uncommon types of uAAs comprises side chains with nucleic bases from DNA or RNA, respectively, which have been successfully incorporated into proteins [147]. For example, MCoTI-II from the squash *Momordica cochinchinensis* is a macrocyclic inhibitor of trypsin, comprising a so-called cysteine knot and a P1-Lys, which was substituted with Guanine-Ala as an isoster of Arg, however, it did not improve the picomolar inhibitory potency of the natural form [148].

#### 3.1.2. Thrombin and Other Blood Coagulation Factors

Based on its crucial role in blood coagulation, thrombin (S01.217) and its major substrate fibrinogen are one of the most studied proteolytic systems [149]. Prothrombin, also known as coagulation factor II, possesses a so-called Gla domain, like several other coagulation factors. The Gla domain contains 10 γ-carboxy glutamates (Gla), which bind Ca^2+^ with high affinity, facilitating the proper function of these coagulation factors. One of the most common chromogenic substrates of the trypsin-like thrombin in enzymatic assays is *D*-Phe-Pip-Arg-pNA (S-2238, Pip is pipecolic acid), which resembles many substrate-like inhibitors, such as the irreversible *D*-Phe-Pro-Arg-chloromethyl ketone (PPACK) or *D*-Phe-Pro-*D*-Arg-*D*-Thr-CONH_2_, inhibiting in the nanomolar range [150]. Measurements of the coagulation reaction for thrombin with fibrinogen by thrombin time (TT) and the thrombospondin time (APTT) confirmed that the inhibitor *D*-Phe-Pro-Orn-(Nδ-SO_2_NH_2_)-OMe significantly prolonged the clotting time [151]. For a combined X-ray crystallographic and NMR study, 5-^19^F-Trp was incorporated into the zymogen prethrombin-2 and mature thrombin by expression in minimal medium, resulting in additional evidence for the conformational selection model of enzyme kinetics with a shifting E*-E equilibrium (Figure 3B) [73,131].

**Figure 4:**
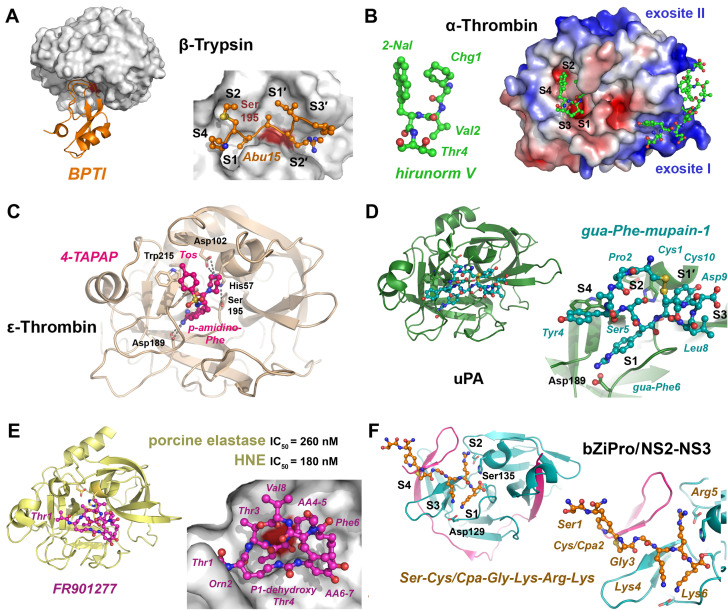
Trypsin-like proteases with various inhibitors. (**A**) Bovine trypsin in complex with a variant of bovine pancreatic trypsin inhibitor (BPTI, PDB 4Y0Z). The protease with the catalytic Ser195 (dark red patch) is depicted as molecular surface and the inhibitor as a secondary structure or with side chains as ball-and-stick models (insert). Abu15 binds to the S1 pocket, and the other subsites are occupied by P4 to P2′ of the inhibitor. (**B**) The complex of human α-thrombin with the engineered hirunorm V (green ball and sticks) essentially shows reversed binding of Chg1 (S2 subsite), Val2 (S1), 2-Nal (S4) and Thr4 (S3). The electrostatic potential at the molecular surface of thrombin is contoured for -5.0 to +5.0 K_B_T/e, whereby the two positively charged exosites are important for protein interactions (PDB 5GDS). (**C**) Active bovine ε-thrombin in complex with the inhibitor 4-TAPAP (tosyl-4-*p*-amidino-Phe-piperidine), which binds to the S4, S1 and S2 pockets. The catalytic triad residues Asp189 and Trp215 are displayed as stick models (PDB 1ETT). (**D**) Complex of uPA with the cyclic mupain-1 (CPAYSRYLDC) variant and 4-guanidino-Phe6, shown as cyan ball-and-stick model that binds to the S1 pocket (PDB 4X1R). The cyclic inhibitor is stabilized by the intramolecular disulfide Cys1-Cys10. (**E**) Porcine elastase (CELA1) and human neutrophil elastase (HNE) are inhibited by the natural bicylic inhibitor *FR901277*. A crystal structure revealed the peptidic linkage of the Orn2 side chain to the ncAA AA6 and the ester bond formed by Thr3-OH and the C-terminal carboxylate of Val8. Another remarkable feature is the P1 residue dehydroxy-threonine, a homolog of dehydroalanine (Dha), with the -CH=CH_2_ side chain (PDB 1QR3). (**F**) A potent inhibitor of the trypsin-like Zika NS2-NS3 protease was formed by cyclization via condensation of an N-terminal Cys with Cpa. The crystal structure of the bZiPro construct revealed the cleaved inhibitor bound to the active site, with the novel uAA (“Cys/Cpa“) occupying the S4 subsite (PDB 6JPW).

As Pro is the preferred P2-residue in thrombin substrates and inhibitors, a large set of P1-Arg-boronate inhibitors was synthesized with Pro analogues, which contained perhydroindole (Phi), azabicyclo[2.2.2]octane (Abo) and azabicyclo[2.2.1]heptane (Abh) or the Pro mimick N-cyclopropylhexyl glycine in P2 position [152]. The corresponding compounds inhibited thrombin and plasmin with IC_50_ values in the low nanomolar and picomolar range. The same P2 screening strategy was applied to neutrophil elastase (HNE, see section below), which prefers small hydrophobic P1 residues, yielding inhibitors with low nanomolar K_i_ values [153]. Further examples of peptidic thrombin inhibitors with nanomolar potency exhibit the basic structure Z-*D*-Phe-Pro-boro-Mpg/Irg, whereby Phe could be replaced by -CH-Phe_2_ side- chains and the boro-moieties by phosphonates, respectively [154]. The highly potent thrombin inhibitor hirudin (I14.001) exhibits a K_i_ value in the low femtomolar range, depending on the source of 1 to 22 fM. Hirudin-derived heptadeca peptides, in which the critical residue Ile59, i.e., position 11, was replaced with *tert*-butylalanine (Tba), decreased the K_i_ from 840 to 390 pM [155]. Similarly, several engineered variants of hirudin, especially the so-called hirunorms IV and V, inhibited thrombin in the picomolar range, due to the presence of Aib, β-Ala, Cha, Nal, *D*-Ala, *D*-Glu and hPhe (Figure 1 and Figure 2) [156]. The crystal structure of human α-thrombin in complex with hirunorm V confirmed that the N-terminus of the inhibitor binds via a reversed backbone as follows: Chg1 (S2), Val2 (S1) and 2-Nal (S4) and Thr4 (S3) (Figure 4B) [157]. Including ncAAs in the natural fragment 1-47 of hirudin by the mutations Val1tBug (*tert*-butylglycine, Tle) and Tyr3-2-Nal decreased the K_d_ (K_i_) for the fast thrombin form from 41 nM to 1.1 nM [158]. Furthermore, the tBug1-Arg2-2-Nal variant replaced the original Val-Ser-Tyr and inhibited the fast (Na^+^ bound) and the slow (Na^+^ free) form of thrombin up to 7000-fold more effectively than the natural hirudin fragment, with K_d_ values of 15.4 pM (fast) and 220 pM (slow), respectively [159].

In addition, the established engineering of hirudin facilitated incorporation of uAAs like 7-azatryptophan and 3-nitrotyrosine (3NY), which reduced the binding affinity for thrombin, but the products were useful as fluorescent probes [160,161]. Rationally designed bivalent thrombin inhibitors targeted the fibrinogen exosite of thrombin and blocked the active site with a non-scissile bond at P1-P1′, based on arginyl ketomethylene isosteres, whereby Xaa as the P1′ residue was Gly, Ala or Pro [162]. Hereby, the most potent compound was *D*-Cha-Pro-Argψ[CO-CH_2_-S]-Gly_5_-Asp-Tyr-Glu-Pro-Ile-Pro-Glu-Glu-Tyr-Cha-*D*-Glu-OH, with a K_i_ value of 350 fM.

Crystal structures of bovine thrombin and the inhibitors NAPAP, 4-TAPAP and MQAP revealed the distinct binding modes of these peptidomimetics, which occupy the S4, S2 and S1 subsites with either naphtyl or tosyl sulfonamide-Gly-(4-amino)Phe/Arg and a piperidine or piperidine-2-carboxylic acid (pipecolic acid, Pip), see Figure 4C [163]. The inhibition constants for human thrombin were roughly 6 nM (NAPAP), 170 nM (4-TAPAP) and 20 nM (MQPA), while the related compound N-α2-naphthyl-sulfonyl-3-amidino-Phe-4-methylpiperidide (*L*-NAPAMP) improved the K_i_ to 2.5 nM [164]. Analogs of the thrombin inhibitor NAPAP, in which the P2-Gly was replaced by uAAs, such as 1-amino-methylglycine (Amg), maintained the potency with a K_i_ of 10 nM and good selectivity among other trypsin-like proteases, and 4-amidino-Phe in P1 was introduced for alkylation extensions [165]. Additional carboxylic groups reduced the systemic and hepato-biliary clearance of similar NAPAP analogs in rats, which may promote pharmacological applications [166]. A study of extended thrombin inhibitor constructs resulted in compounds such as dansyl-Arg-*D*-Pip (P4–P1), β-Gly-Gly-Gly-*D*-amino-pentanoic acid-(P1′-P4′)-hirudin-55-65 (exosite I) or µ-amino dodecanoic acid in the P1′–P4′ stretch, which all inhibited in the lower picomolar range [167,168]. A prime side screening with sarcosine (Sar), *D*/*L*-Ala, *D*/*L*-3-aminoisobutyric acid, N-methyl-a-Ala and Cha improved the inhibitory potency of the resulting Bbs-Arg-(*D*-Pip)-[linker]-DYEPIPEEA-Cha-*D*-Glu, with a phenyl ring and ζ-amino heptanoic acid as the linker main chain [169,170]. The compound inhibited thrombin with a K_i_ of 230 fM, relatively close to the potency of hirudin with a K_i_ of at least 22 fM. Another synthetic compound, *D*-Arg-Oic-Pro-*D*-Ala-(p-Me)F-NH_2_ (FM19), showed promising effects in platelet inhibition of acute coronary syndromes associated with diabetes, despite being a low-affinity inhibitor of thrombin (Figure 2) [171]. The X-ray crystallographic structure corroborated a reverse binding mode, where the *D*-Arg residue of *FM19* binds to the S1 pocket.

Aeruginosins are natural small-molecule inhibitors of the coagulation factors thrombin and factor VIIa, which contain a D-amino acid in the P3 position and inhibited in the nanomolar range. However, a D-P3 residue usually occupies the S4 subsite of trypsin-like serine proteases, whereby the strongest inhibition of thrombin was observed for a hybrid aeruginosin with a corresponding “P3” *D*-3R-chloroleucine with an IC_50_ of 1.6 nM [172]. Sub-picomolar inhibition was found for the 32-residue-long tsetse fly thrombin inhibitor (TS), which was screened for sulfo-Tyr (sY) variants, yielding (N-Me)L32-sY9/sY12-TTI with a K_i_ of 60 fM [173].

A study on inhibitors of the urokinase-type plasminogen activator (uPA, S01.231) showed that the murine counterpart of upain-1, the cyclic mupain-1 (CPAYSRYLDC) with *L*-4-guanidino-cyclohexyl-Ala instead of P1-Arg6, inhibited murine uPA 10-fold better with a K_i_ of 45 nM [174]. A similar screening of mupain-1 was based on a phage-display peptide library with P1-Arg6 analogs, such as 4-guanidino-Phe or F(gua), which improved the K_i_ to about 185 nM for the variant position, supported by the Asp9Ala mutation (Figure 4D) [175]. Activated protein C (APC), thrombin and factor Xa were screened for the P4–P1 specificity with about 50 AAs, of which the unnatural ones often resembled the natural ones [176]. Based on these results, fluorescent substrates of the type of Ac–Lys–Dab(Z)–Igl–Arg–ACC reached catalytic efficiencies of 71,000 up to 8,700,000 M^−1^ s^−1^, whereby Dab(Z) and Igl are benzyloxycarbonyl-*L*-2,4-diaminobutyric acid and *L*-2-indanyl-glycine. Subsequently, the corresponding ABPs allowed to detect and to quantify their target protease from the coagulation cascade in human plasma. A similar approach resulted in the first fluorescent ABP that is selective for factor XIa over other coagulation proteases, namely, Bodipy-PEG4-Tyr(2,6-Cl_2_-Z)-Nle-Glu(Bzl)-ArgP(OPh)_2_ [177]. As blood coagulation factors and related proteases are favored targets for ABPs, the factor VII activating protease could be detected in human blood plasma by Cy5-ε-Ahx-Pro-*D*-Tyr-Lys-Arg-P(OPh)_2_, with the fluorescent cyanine5 group (Cy5).

A de novo design study was based on a DNA library with a random sequence of ten residues encoding at least three uAAs cyclized by two Cys residues. These ncAAs included homopropargylglycine (Hpg), canavanine (Can), 4-Bromo-Phe, a Lys analog with a double bond between Cγ and Cδ, tBu-Gly (Tle) and a Pro with a sulfur in γ-position. K_i_ values for thrombin inhibition ranged from 6 to 35 nM for the three best cyclic inhibitors, which comprised a linker and a His_6_ tag [178].

#### 3.1.3. Kallikrein-Related Peptidases, Cathepsins, Neutrophil Serine Proteases and Tryptases

Kallikrein 1 (KLK1, formerly hK1, S01.160), the “true tissue kallikrein”, has a dual chymotryptic/tryptic specificity for P1-Tyr/Arg [164,179]. Surprisingly, the screening for substrates with basic ncAAs in the P1- and P3-positions resulted in KLK1 inhibitors of the nanomolar range, exhibiting *trans*-4-aminomethylcyclohexylalanine (Ama) in Abz-F-Ama-S-R-Q-EDDnp, whereas variations of basic and hydrophobic uAAs in P3 resulted in phenylacetyl-Aca-Ser-Arg-NH_2_ as the best inhibitor (Aca is *trans*-4-aminocyclohexylalanine, Figure 2) [180,181]. Kallikrein-related peptidase KLK2 exclusively prefers basic P1 residues, as corroborated by the best fluorogenic substrate Ac-Orn-Phe-Arg-AMC, with the shorter Lys homolog ornithine (Orn) in the P3 position [182]. The chymotryptic PSA or kallikrein-related peptidase 3 (KLK3, S01.162) is a major target of biochemical and pharmaceutical research, because it is a critical factor in prostate cancer. Screening of P2 and P3 libraries with ncAAs, such as Cha, Hse, Met(o), Nle, Nva, Phg and Nal, yielded peptidomimetic aldehyde inhibitors like Z-SSK-X-L-al and boronic compounds like Z-S-(N-Me)SKLL, with a K_i_ value of about 200 nM [183]. Similar to tissue kallikrein KLK1, plasma kallikrein (KLKB1, S01.212) regulates the blood pressure by releasing bradykinin from kininogens. Moreover, it is involved in the contact activation system by activating factor XII, which subsequently initiates the intrinsic blood coagulation pathway, and it is a suitable target in diabetes-related pathologies [184]. A complex cyclic peptide with ncAA substitutions, such as hArg, Aze, Ala(ψCH_2_NH) and three tribromomethylbenzene-linked Cys residues, inhibited KLKB1 with a K_i_ of 360 pM and it had positive effects in rodent diabetes model systems.

Human cathepsins comprise two serine proteases (CatA, S10.002 and CatG, S01.133), of which cathepsin G is more important due to its role in the neutrophil leukocytes’ mediated immune response [185]. A study on the human chymotrypsin-like CatG may serve as a major example for the usage of ncAAs in chromogenic substrates. As aliphatic and aromatic side chains such as Leu, Phe and His are preferred in P1 by CatG, a tetrapeptide library with 5-amino-2-nitrobenzoic (Anb) as chromophore was generated, with the general formula Ac-Phe-Val-Thr-Xaa-Anb [186]. The substrate with 4-guanidino-Phe F(gua) in P1 had a catalytic efficiency (k_cat_/K_M_) ten times higher than the second best 4-amino-Phe, which was still preferred over P1-Tyr and Phe. The already mentioned sunflower trypsin inhibitor (SFTI-1) is one of the most frequently engineered model systems that was adapted to many serine proteases, such as chymotrypsin and CatG, which were strongly inhibited by P1-Arg5 variants with para-substituted Phe, e.g., F, NO_2_, NH_2_, Me and Gua, according to the association constant K_a_ [187,188]. Matriptase-1 (S01.302) and Matriptase-2 (S01.308) are cancer- and iron homeostasis-related type II trans-membrane serine proteases with a strong preference for P1-Arg. A systematic screening of uAAs in positions P4–P2 of inhibitors with a ketobenzothiazole serine trap moiety in the P1′ position resulted in a compound with a K_i_ of 4 nM for matriptase-1 and 400 pM for matriptase-2 [189].

The optimization process for fluorogenic FRET or IQF substrates in the P1′ to P3′ positions in a comparative study of the serine proteases cathepsin G (CatG) and human neutrophil elastase (HNE, elastase-2), proteinase 3 (PR3) and “non-structural protein 4” (NSP4) found the most potent substrates exhibiting Nva in P1′ and 3-Pal or Met(o) in P3′ [190]. Previously, it was observed that the most efficient ABP for CatG contained a P1-F(gua), whereas HNE reacted only with a P1-Leu probe [191]. As HNE levels rise in blood upon microtrauma, plasma and in vitro stability was tested for decapeptides with ethylglycine, 4,4-difluoroethylglycine (DfeGly) and 4,4,4-trifluoroethylglycine (TfeGly) in positions 4, 6 and 7 (Figure 2) [192,193]. A study with the same setup demonstrated that peptides with these ncAAs in the P2, P1′ and P2′ positions were better substrates for chymotrypsin and pepsin [194]. A large hybrid combinatorial study substrate library (HyCoSuL) with 102 uAAs was employed to explore the S1–S4 pockets of HNE, resulting in the ideal substrate Ac-Nle(OBzl)-Met(o)_2_-Oic-Abu-ACC and the corresponding ultrasensitive ABP *PK101* [195]. The corresponding ABP, namely, biotin-PEG4-Nle(O-Bzl)-Met(o)_2_-Oic-Abu-PO_3_Ph_2_, was crystallized in complex with HNE and revealed details of the interaction with the S4–S1 pockets, e.g., exosite binding beyond S4 [196]. *FR901277* is a natural bicyclic inhibitor of elastases isolated from Streptomyces resistomicificus, with an IC_50_ of 180 nM for HNE and 260 nM for porcine elastase (Figure 4E) [197]. The sequence can be described as Thr-Orn-Thr-dehydroxy-Thr-AA4-Phe-AA6-Val, whereby AA4 and AA6 are complex ring-containing amino acids, with additional unusual ester and amide bonds formed from the Thr-OH to the Val8 carboxylate and from the Orn2 εNH_2_ to a carboxylate of AA6. A high-resolution X-ray structure confirmed that the compound binds from the S4 to S2′ pockets of porcine elastase, with dehydroxy-Thr as the P1 residue. Focusing on NSP4, the optimized substrate Ac-hCha-F(gua)-Oic-Arg-ACC was obtained, as well as the highly specific ABP biotin-ε-Ahx-hCha-F(gua)-Oic-ArgP(OPh)_2_ (Figure 2) [198]. Based on a HyCoSuL, highly selective ABPs for cellular assays were developed, in order to target the four neutrophil serine proteases HNE, CatG, PR3 and NSP4 [199]. For another trypsin-like serine protease from leukocytes—granzyme B from natural killer cells—optimal substrates were generated, which contained ncAAs in positions P5 to P2 such as Tic, Oic, Hyp, Tyr(Bzl) and others, which subsequently allowed to synthesize specific ABPs [200].

Human mast cells secrete both the trypsin-like α-tryptase (S01.143) and the more abundant β-tryptase (S01.015) as part of distinct immune responses, whereby mainly β-tryptase forms tetramers upon activation [201]. As tryptases are involved in inflammation and allergy, inhibitors could be valuable pharmaceutical compounds, such as cyclotheonamides from the marine sponge species *Ircina* [202]. The cyclotheonamides E and E4 inhibit human tryptase, with IC_50_ values around 5 nM, and the E5 variant inhibits at 85 nM. Both contain the unusual ncAA with an amino-methyl side chain and a Tyr derivative with a CH=CH group inserted between the Cα and the carbonyl C.

#### 3.1.4. Viral Serine Proteases

Flavivirueses carry a single-stranded RNA and cause various diseases, such as Dengue fever, Yellow fever or European tick-borne encephalitis, which causes up to 80,000 deaths per year worldwide [203]. Therefore, the crucial major protease flavivirin (S07.001) from the trypsin-related flaviviral serine proteases is a target for drug development. In a comprehensive study of the dengue virus (DENV) NS2B-NS3 protease, fluorescent substrates of the type Bz–Xaa-Arg–AMC enhanced the catalytic efficiency with Xaa = Amf (4-aminomethylphenylalanine), Ama and Aca compared to Arg [204]. With respect to the canonical inhibitor sequence Bz-Arg-Lys-Nle-NH_2_ for dengue virus serotype 2 (DENV-2) protease, a substitution of the Bz cap with a more complex residue and a P1′-Phg decreased the K_i_ to 400 nM [205]. An optimization of DENV protease inhibitors in the P3 and P2 positions yielded trifluoromethyl-Bz-4-amidino-Phe-Lys-Phg-OH with an IC_50_ of 210 nM [206]. Systematic screening for cap (P3) and P1′- Phg derivatives around the P2-Arg-P1-Lys core of DENV-2 and West Nile virus (WNV) proteases resulted in compounds with thiophene or thiazole caps and P1′-4-hydroxy-phenylglycine ethers, such as Phg-O-4-CF_3_-Bz, which reached K_i_ values of 12 nM (DENV) and 39 nM (WNV), whereby virus replication was also inhibited [207]. Substrates with ncAAs were analyzed for the NS2B-NS3 proteases of DENV, WNV and Zika virus (ZIKV) and allowed to synthesize corresponding ABPs [208]. As D-amino acids are favored over L-enantiomers in P4, the specific ZIKV NS2B-NS3 protease substrate Ac-*D*-Arg-Lys-Orn-Arg-ACC was generated as well as the corresponding phosphonate inhibitor/ABP biotin-ε-Ahx-*D*-Arg-Lys-Orn-Arg-P(OPh)_2_ [209]. Apart from Arg, Arg(Me)_2_ was the most specific P1 residue for all these flaviviral proteases, whereas P2-Orn and hLys (DENV) were preferred, and *D*-Arg or *D*-Leu (DENV) were favored in P4. Derived from a substrate of the Zika virus protease, a peptide with an N-terminal Cys and a C-terminal 3-(2-cyano-4-pyridyl)alanine (Cpa) cyclized spontaneously to a dihydrothiazole and yielded an inhibitor of proteolytic stability and high affinity for the NS2B-NS3 bZiPro construct with a K_i_ of about 140 nM. However, the linear form of the inhibitor was observed in a complex bZiPro crystal structure, with the novel uAA “Cys/Cpa” binding to the S4 pocket and beyond (Figure 4F) [210].

The related hepatitis C virus (HCV) comprises the NS2 cysteine protease CPro-1 (C18.001), which directly precedes the NS3 serine protease (hepacivirin, CPro-2, S29.001), with a Zn^2+^ contributing to autoproteolysis of the viral polyprotein [211]. The P1 specificity was investigated for the NS3 domain with and without the NS4 activator peptide, using substrates that contained both cAA and ncAA residues like hCys, Abu, and Alg, nearly reaching the catalytic efficiency of P1-Cys substrates [212]. In a similar study, substrates with ncAAs derived from the viral NS5A/B protein showed no improvement but generated the H_2_N-EDVLC-Tic-Nle-SY-OH inhibitor (Tic: 1,2,3,4-tetrahydroiso-quinoline-3-carboxylate) with a K_i_ of 340 nM [213]. A purely computational QSAR screen for ncAAs in hexapeptides, docked to the S6-S1 subsites of hepacivirin, predicted inhibitors with subpicomolar Ki values, namely, Ac-Asp-*D*-Gla-Trc-Asp-Asp-Fab/Cyo, which exhibited 4-carboxy-Trp (Trc), α-keto-δ,δ-difluoro-aminobutyric acid (Fab) or α-ketocysteine (Cyo) [214]. Extensive screening for the P3 to P2′ positions of peptidomimetic inhibitors with Chg, Nva or Phg, yielded a best K_i_ of 12 nM, and an X-ray structure of an NS3/4a protease complex gave further insights into details of inhibitor binding [215]. Several inhibitors with low nanomolar potency consist of uAAs, such as Pro derivatives with isochinoline ethers as substituents at the Cγ or a residue with a vinyl-cyclopropyl side chain, which covalently links to the non-catalytic Cys159, achieving an IC_50_ of 2 nM [216]. Proline derivatives fused to C60 fullerenes at the Cβ and Cγ atoms with a carboxylate substitution at the Cδ atom inhibited the HCV NS3/4A serine protease with an IC_50_ of 150 nM, making them promising virostatic lead compounds [217]. After successful clinical phase III trials, the low nanomolar inhibitor of hepacivirin, Voxilaprevir, was approved by the US Food and Drug Administration (FDA) for treatment of patients [218]. It features a Tle residue and three cyclopropane groups, among them an uAA with a difluoromethylcyclopropyl moiety, including the Cα atom.

#### 3.1.5. Subtilisin-like Serine Proteases and α/β-Hydrolases

An early screening study with ncAA containing inhibitors of the P1-Arg-specific furin (S08.071) and the subtilisin-like proprotein convertase-1 (PC1, S08.072) utilized the sequence *D*-Tyr-Arg-Ser-Lys-Arg-Xaa-Val-Gln-Lys-Asp, whereby Xaa in the P1′ position was various ncAAs [219]. Inhibition of both proteases occurred with γ-Abu, β-Cha and β-Ala as Xaa, which resulted in a nanomolar K_i_ for furin with the β-Ala derivative. Essentially, this project was extended by using, e.g., Tle, Sar, MLeu, Aib, *D*-Tic, and *L*-Tic demonstrating that inhibition of furin was more efficient with P1′ Tle (*tert*-butylglycine) (Figure 1 and Figure 2) [220]. A more potent inhibitor of furin was 4-guanidinomethyl-phenylacteyl-Arg-Tle-Arg-4-amidinobenzylamide, with a K_i_ value of 5.5 pM [221]. Substrate-derived compounds with aminooxy-acetic acid (Aoaa) or 8-amino-3,6-dioxa-octanoic acid (Adoa) in the P1 and P1′ positions inhibited the subtilisin kexin isoenzyme I (S08.063) from the proprotein convertase family in the low micromolar range [222]. Semisynthetic variants of the inhibitor eglin C (I13.001) obtained by native chemical ligation, phage display and stop codon suppression for the incorporation of kynurenic acid (Kyn) were captured by subtilisin BPN′ (S08.034) for selection [223]. SufA (S08.138) from the subtilisin-like serine protease family hydrolyzes human fibrinogen during infections with the gram-positive bacterium *Finegoldia magna*, which motivated substrate screening of uAA P1-Arg analogs that led to the synthesis of the inhibitor Cbz-6-AmNphthP(OPh)_2_ with micromolar potency [224].

**Figure 5:**
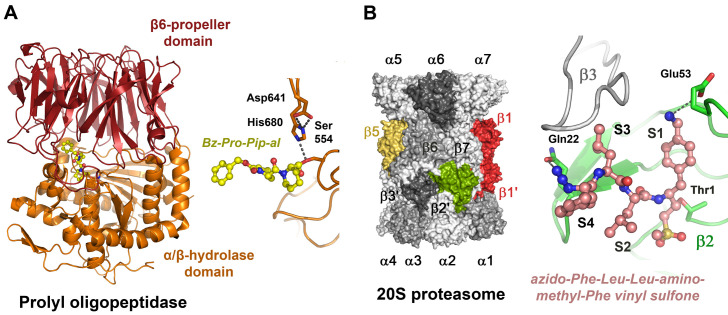
Compartmentalized serine and threonine proteases. (**A**) Prolyloligopeptidase active site with the aldehyde inhibitor Cbz-Pro-formyl-piperidine, an imino acid (PDB code 2XDW). The covalently bound inhibitor occupies the S3 to S1 subsites (yellow ball-and-stick model), and the left panel displays the propeller domain (red) and the peptidase domain (orange). The catalytic triad (Ser554, Asp641, H680) is shown as sticks in the right panel. (**B**) The eukaryotic 20S core of the proteasome consists of two rings of seven α subunits and two rings of seven β subunits, with the active β1 (PGPH, caspase-like), β2 (green trypsin-like) and β5 (yellow, chymotrypsin-like) subunits (left panel).The synthetic inhibitor (salmon ball-and-stick model) occupies specificity pockets S1 to S4, with the ncAA aminomethyl-Phe mimicking a P1-Arg or Lys. A covalent bond is formed between the vinyl sulfone and the catalytic Thr1 (PDB code 4INR). Such compounds enhance the effects of anti-cancer drugs, e.g., bortemozib, which mainly target the β5 active sites (right panel).

Prolyl oligopeptidase (POP, prolyl endopeptidase, S09.001) belongs to the α/β-hydrolase family and exhibits a large propeller domain, which forms a compartment that harbors the active site. It cleaves after P1-Pro, whereby a marked preference for Pro in positions P4, P3, P1′ and P3′ was observed [225]. Several POP keto heterocyclic inhibitors have been developed based on analogs of the central P1-Pro, such as thioproline (Thp), Phi, Abo and Abh, resulting in IC_50_ values in the low nanomolar range (Figure 2) [226]. The synthetic inhibitor benzylcarboxylate-Pro-2-formylpiperidine was crystallized in a complex with human POP containing an unnatural imino acid derivative (Figure 5A) [227].

Quite unusually, penicillin-binding proteins (PBPs) with peptidase activity can cleave peptidoglycans at positions with D-amino acids, resulting in biofilm formation. Among them are PBP4, PBP4a and PBP5 from *E. coli* and *Bacillus* subtilisin (*D*-Ala- *D*-Ala peptidases S13.001/2 and S11.001/8), respectively, which also cleave at *D*-Asn, *D*-His, *D*-Trp and various other ncAAs [228]. General inhibitions of PBP protease activity have been achieved by incorporating ncAAs into pharmaceutical peptides for better efficacy by increasing intracellular concentrations [69,229]. Lactoferrin (S60.001) is first and foremost an iron transport protein that, surprisingly, can function as a protease with trypsin-like specificity, cleaving substrates such as Z-Phe-Arg-AMC [230].

### 3.2. ATP-Dependent Proteases

Self-compartmentalizing proteases perform important tasks in the degradation of misfolded proteins in all organisms and in the immune system of higher organisms, with the 26S proteasome as the most prominent example of the N-terminal nucleophile (Ntn) hydrolases with a catalytic Thr1 [231]. In addition, the ATP-dependent ClpXP-complex exists in many bacteria and human mitochondria, whereby ClpP (caseinolytic protease P, S14.003) exhibits a chymotrypsin-like P1 specificty, whereas ClpX is an AAA-ATPase/unfoldase. Based on a HyCoSuL profiling, the ideal substrate Ac-Phe(3,4-Cl_2_)-hArg-Leu-ACC for *Staphylococcus aureus*, *E. coli* and human ClpP protease was obtained [232]. A related study screened many P3 to P1 residues, including uAAs, and established that 2,5-dichlorobenzoyl-Trp-Leu-boronate inhibits ClpP of *S. aureus* only in the nanomolar range [233]. In an indirect manner, natural cyclopeptidic inhibitors of the ClpC1 unfoldase from *Mycobacterium tuberculosis* can effect a completely uncontrolled protease activity of ClpP that kills the mycobacterial cells [234]. These protease activity-stimulating compounds, e.g., ilamycins, rufomycins, and cyclomarins from marine *Streptomycetes* contain unusual ncAAs, such as nitro-Tyr, various Nε1-substituted Trp or isopentanol and butene side chains.

Lon or PIM1 is a highly conserved serine protease (S16.002) present in all domains of life. It consists of a substrate-binding domain, a serine protease domain, and an ATPase domain on a single polypeptide chain, which forms hexamers and mainly degrades misfolded proteins [235]. A HyCoSuL screening for *E. coli* Lon specific substrates resulted in the potent covalent boronate inhibitor Pyz-hArg-NptGly-Leu-B(OH)_2_ with a k_inact_/K_I_ of 3400 s^−1^ × M^−1^, which resembles the proteasome inhibitor bortemozib, but confers higher selectivity for Lon over the proteasome (NptGly is neopentylglycine) [236].

The eukaryotic proteasome with the active subunits β1, β2 and β5 (T01.010/11/12) and its bacterial counterparts such as HslVU (ClpQ, T01.006) in *E. coli* are the most widespread and important representatives of this protease class. Although the ubiquitin-26S proteasome machinery is crucial for protein degradation, immuno- and thymo-proteasomes fulfill additional tasks [237]. Dysregulated proteasomes are associated with cancer, which makes them suitable targets for pharmaceutical inhibitors that can selectively bind to the active sites of the β1 (PGPH, caspase-like specificity for Asp and Glu), β2 (trypsin-like) and β5 (chymotrypsin-like) subunits. A study on internally quenched fluorescent (IQF) substrates with both ncAAs and uAAs in the P1′ and P2′ positions for the 20S proteasome found an increased specificity for the chymotrypsin-like subunit in comparison to shorter tetrapeptides [238]. Both fluorescent substrates and ABPs for distinct protease subunits of the 20S core particle in human cell lines contained various ncAAs in the P1 position and facilitated to monitor the activity and localization of proteasomes [239]. However, the human proteasome is an important target in cancer therapy because inhibition of its chymotryptic activity results in a highly selective cytotoxicity to cancer cells [240]. Resembling chymotryptic substrate stretches, new CBZ-AzBzH-Ile-Leu-boronate-Phe/Leu (AzBzH, (S)-6-Azido-2-(benzyl-oxy-carbonylamino)-hexanoic acid peptidomimetics were synthesized that had lower IC_50_ values than the pharmaceutical drugs bortezomib and carfilzomib, which contains an hPhe. Nevertheless, inhibitors of the trypsin-like proteasome activity serve as enhancers or sensitizers of bortezomib and carfilzomib in the treatment of cancer, and thus the highly potent inhibitor α-azido-Phe-Leu-Leu-4-aminomethyl-Phe-methyl vinyl sulfone was developed and structurally characterized as *Saccharomyces cerevisiae* 20S complex (Figure 5B) [241]. Although they are not ATP-dependent and are classified as a mixed protease family (P01), the octameric β-aminopeptidases BapA from *Sphingosinicella xenopeptidilytica* and DmpA from *Ochrobactrum anthropi* belong to the Ntn hydrolases with an αββα architecture like the proteasome [242]. Both BapA and DmpA cleave ncAAs with an N-terminal β-amino group, i.e., β2AAs with a side chain R linked to the Cα and β3-AAs with the side chain R at the Cβ [243]. BapA and DmpA hydrolyzed dipeptides such as H-β2hPhe-β2hAla-OH, which was accompanied by a diastereomeric conversion of β2hAla, but they also catalyzed the ligation of oligopeptides, such as H-[β2hPhe]3-β2hAla-OH.

### 3.3. Cysteine Proteases

Most cysteine protease contain a catalytic triad or dyad, with a nucleophilic Cys and an activating, stabilizing His [24]. As already mentioned, human cathepsins comprise serine proteases, as well as aspartic proteases (CatD, CatE) and 11 cysteine proteases [185]. Cathepsin B (CatB, C01.060) is related to the prototypical papain (C01.001) and is a major drug target, as it is involved in Alzheimer’s disease and various cancer processes. Therefore, a recent study on CatB was performed with a library containing many ncAAs, in order to obtain optimal substrates and fluorescent ABPs [244]. Surprisingly, the most preferred P1 residues of CatB were exclusively uAAs, such as Lys(2Cl-Z), Cys(Bzl), Cys(MeBzl), Cys(Me)Bzl and Nle(OBzl), as well as hSer(Bzl) in P2, Phg in P3 and hCha in P4. Based on these results, ABPs for cathepsins B, L, V and S were obtained and tested in cancer cell lines. An emerging field in this context is the investigation of protease activity in cells and organisms or tumor tissue extracts with the HyCoSuL approach, which was applied with ABPs for cathepsins B, S and L [245]. Cathepsin C or dipeptidyl peptidase I (CatC, DDPI, C01.070) is a lysosomal papain-like cysteine protease and an important activator of other proteases with functions in the immune system and inflammation. A study of human, bovine and malarial CatC with a natural P1-Arg and P2-Met preference revealed that 4-benzoylphenylalanine (Bpa) and Nle(OBzl) are essentially more favorable in the P1 position, and Hse and Abu in P2, whereas Pip is the preferred P2 residue in malarial CatC [246]. Screening of P1 and P2 specificity in papain-like proteases with basic uAAs led to the synthesis of better substrates with respect to P2-Phe/Arg-P1-Arg for cysteine protease B (CPB) and cruzaine from *Leishmania mexicana* and *Trypanosoma cruzi* parasites, respectively, as well as for cathepsin L (CatL) and the eponymous papain from *Carica papaya* [247,248]. By contrast, CatB, a rather unspecific protease with a preference for P1-Gly, turned over ε-Ahx-Leu-Thr(OBzl)-AMC at a high rate, like papain, which favored a P1-Cys(SBzl) [249]. The cytosolic self-compartmentalizing bleomycin hydrolase (C01.084) is relatively unspecific but a target for cancer therapy, for which the substrates Lys(2-Cl-Cbz)-ACC and Cys(Bn) and the corresponding vinylsulfones ABPs have been developed [250].

The C2 family of cysteine proteases comprises the calpains, e.g., calpain-I (C02.001), whereby a Ca^2+^ regulates the activity of the multisubunit calpastatins (I27). In a comparative study of calpain-1/2 (µ/m) and cathepsins B, K and L, epoxide compounds with low nanomolar potency were developed, whereby the two best inhibitors, *WR13* and *WR18*, exhibited Alg in the P2-position and γ-cyano-α-aminobutyric acid [251]. In order to assess the role of the rotamers for distinct residues at the calpain–calpastatin interface, more than 100 uAAs, such as Amt (α-methyl-tryptophan) and 4-methyl-Phe, including the natural ncAA Sec and others, were modeled using the ROSETTA3 software [252].

**Figure 6:**
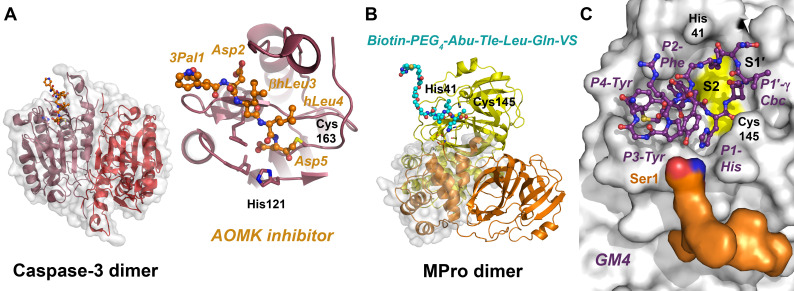
Cysteine proteases in complex with ncAA-containing compounds. (**A**) Similar to other caspases, human Casp-3 is active as a symmetrical dimer. Ac-3Pal-Asp-*D*-βhLeu-hLeu-Asp-acyloxymethyl ketone (AOMK), depicted as ball and sticks, inhibits Casp-3 with an IC_50_ of 23 nM and binds covalently to the catalytic Cys163 near the dyad residue His121, occupying the S5 to S1 specificity pockets (PDB 4JJE). (**B**) The viral SARS-CoV-2 MPro is a cysteine protease with a chymotrypsin-like fold. MPro is also active as a dimer, which is stabilized by the so-called domain III (with transparent surface), and the protease consists of the two half domains I and II. Covalent binding to the catalytic Cys145 was established by the efficient activity-based probe Biotin-Peg_4_-Abu-Tle-Leu-Gln-vinyl sulfone [265], shown as a spherical model. (PDB 6Y2E). (**C**) *GM4* is a tricyclic compound with His-P1 and comprises a γ-amino acid cis-3-aminocyclobutane carboxylic acid in the P1′ position that inhibits MPro at a K_d_ value of 5.2 nM. A crystal structure revealed the binding mode from the S4 to S1′ pockets (PDB 7Z2S) The catalytic dyad is displayed as yellow patches on the molecular surface of MPro. The N-terminus of the dimer counterpart molecule (orange surface) contributes mainly with Ser1 to the S1 and S3 subsite, and vice versa.

Caspases are dimeric cysteine proteases (family C14), which often cleave after the consensus sequence DXXD (P4-P1). Typically, fluoromethyl ketones are the classical covalent caspase inhibitors, e.g., Cbz-Val-Asp-FMK, which was used as a starting model for the incorporation of 2-aminobenzoic acid (anthranilic acid, 2AA) derivatives, yielding Cbz-Val-2AA-Asp-FMK, which inhibited caspases 1, 3, 6, 7, 8 and 9, with IC_50_ values in the low nanomolar range [253]. A comparative study of caspase-3 and 7 inhibitors, as well as ABPs, demonstrated that DEVD was a discriminating sequence [254]. However, one of the most potent inhibitors of Casp3, with an IC_50_ of 23 nM, was Ac-3Pal-Asp-*D*-βhLeu-hLeu-Asp-AOMK (acyloxymethyl ketone, 3Pal is 3-pyridyl-alanine), consisting altogether of three ncAAs. This compound was the basis for the synthesis of carboxyfluorescein (FAM) and biotin-tagged ABPs (Figure 6A). As the Ac-LETD-AOMK inhibitor is very potent, but does not discriminate between caspases 3, 6, 7, 8 and 9, a HyCoSuL screening revealed the compound β-Ala-Leu-Glu-Hyp-Asp-AOMK, which was highly specific for Casp8 and Casp9 [255]. Similarly, a comparative HyCoSuL study of Casp-3, 6, 7, 8, 9 and 10 for the P4–P2 positions found very selective substrates for caspases 8, 9 and 10, such as Ac-*D*-hPhe-Aad-Thr(Bzl)-Asp-ACC, Ac-Oic-Tle-His-Asp-ACC and Ac-Nle(OBzl)-Dab-Nle-Asp-ACC [256]. Internally quenched fluorescence (IQF) substrates with the fluorophore ACC and N(6)-(2,4-dinitrophenyl)lysine (Dnp-Lys) as the quencher exhibited an enhanced signal in specificity profiling of caspases 3, 7, 8 and 9, as well as of HNE, legumain and MMPs 2 and 9 [257]. MALT1 is the only human paracaspase (C14.026) with a unique preference for P1-Arg. In order to obtain potent ABPs, libraries with fluorogenic substrates were screened for the P5–P2′ positions, resulting in an idealized recognition sequence F(gua)-LVSR↓GT/Abu and, consequently, in the ABP F(gua)-LVSR-AOMK with an acyloxymethyl ketone warhead [258]. A related study improved corresponding substrates and ABPs with respect to their selectivity for MALT1 by reducing the cross-reactivity with CatB, whereby a P2-Pip was highly critical [259]. Legumain or asparagine endopeptidase (AEP, family C13.004) has a similar fold as caspases, but it is active as a monomer [260]. Employing aza-peptidyl Michael acceptor and expoxide warheads, a cyclopropyl-uAA-Pro compound was discovered that inhibited legumain, with an IC_50_ of 4 nM, and Cy5-linked counterparts are highly selective ABPs for legumain.

Based on a HyCoSuL P2 screen with many ncAAs, the substrate specificity profile for two ubiquitin-specific peptidases (DUBs), MERS papain-like protease (PlPro, C16.011) and human UCH-L3 (C12.003) was determined, which allowed to generate ubiquitin-based substrates and ABPs containing suitable ncAAs positioned in the C-terminal Ub motif [261]. Autophagins such as ATG4 proteases (family C54) process Atg8 proteins for their conjugation to the membrane component phosphatidylethanolamine, which results in autophagosome formation, as well as for their subsequent deconjugation by SENPs (family C48). Using a positional substrate scanning library (PSSCL) for Atg4 of *Trypanosoma crucei* of the type Ac-Xaa-Xaa-Cha-Gly-ACC allowed to distinguish it from human Atg4, SENP1, SENP2 and UCH-L3 [262]. The parasite *Plasmodium falcipare* possesses the papain-like falcipains 2 and 3 (C01.046/063) as key targets for anti-malarial drugs [263]. In a study that included the related trypanosomal rhodesain (C01.072) inhibitors with a central Michael system (CO-CH_2_=CH_2_-CO) and mostly Chg and Phg in the P2 position, the most potent compounds had K_i_ values around 400 nM. A screening for about 20 cinnamic acid variants as C-terminal caps was undertaken for a hPhe-Leu dipeptide core with 4-aminoquinoline, a component of chloroquine (CQ) as an N-terminal cap [264]. Some of these compounds showed potency against the proteases in the lower µM range and against the CQ-resistant *P. falciparum* strain W2.

Not surprisingly, the SARS-CoV-2 corona virus during the COVID 19 pandemic has stimulated many academic and pharmaceutical studies to explore and develop peptidomimetic inhibitors of the main protease MPro of SARS-CoV-2. This cysteine hydrolase is also termed 3-chymotrypsin-like protease (3CLPro, C30.007), exhibiting a preference for P2-Leu and P1-Gln residues as an active dimer [266]. A recent approach with variations in the P2 and P3 positions, comprising mostly ncAAs, yielded 18 synthetic tripeptidyl MPro inhibitors with the aldehyde warhead β-(S-2-oxopyrrolidin-3-yl)-alaninal at the P1 position, whereby a P3 O-tBu-Thr conferred the highest potency [267,268]. The best compound reached nanomolar values for IC_50_ in vitro, as well as for cellular and antiviral EC_50_, and crystallographic data corroborated the mode of binding and inhibition. Proline derivatives merged with C60 fullerenes at the Cβ and Cγ atoms and substituted with a carboxylate at the Cδ atom inhibited MPro with IC_50_ values around 1 µM, while a malonic acid fullerene fusion molecule exhibited an IC_50_ of 200 nM [269]. Also, acyloxymethyl ketones as ABPs for the main cysteine-type protease of SARS-CoV-2 were developed, with the P4–P1 sequence Abu-Tle-Leu-Gln/His, which was additionally modified with P2-Cha, P1-Cit and GlnMe_2_ [270]. Based on a comprehensive substrate profiling, the most favored Ac-Abu-Tle-Leu-Gln-ACC and the irreversible SARS-CoV-2 MPro inhibitor Ac-Abu-*D*-Tyr-Leu-Gln-vinyl sulfone were synthesized, as well as corresponding ABPs with a PEG linker and a biotin moiety (Figure 6B) [271]. Moreover, the crystal structure of an ABP-MPro complex was determined, and active MPro was visualized in nasopharyngeal epithelial cells of patients with COVID-19 infection. A structure-based study combined with virtual screening in the AutoDockFR (AutoDock4, v4.2.6) software was successful, followed by a molecular dynamics simulation with GROMACS 2018.6. Eventually, chloro-acetamide-based compounds were obtained with electrophilic warheads that had low micromolar affinities [265]. The flexible in vitro translation (FIT) system allows to reprogram the genetic code with so-called flexizymes, which are flexible tRNA acylation ribozymes that facilitate the preparation of acyl-tRNA libraries with all types of ncAAs and the ability to read liberated codons for the synthesis of unusual peptides [272]. Based on this method, cyclic γ2,4-peptides with up to 16 residues and different uAAs were prepared, to find potent SARS-CoV-2 MPro inhibitors [273]. In particular, His-P1 and the cyclic γ-amino acids cis-3-aminocyclobutane carboxylic acid (γ1) and (1*R*,3*S*)-3-aminocyclopentane carboxylic acid (γ2) in the P1′ position enhanced the potency of these inhibitors with low nanomolar IC_50_ values and a best K_d_ below 1 nM. The binding mode of the cyclobutane-containing inhibitor *GM4* with a K_d_ of 5.2 nM was analyzed in more detail in a high-resolution crystal structure (Figure 6C).

A less-studied viral protease is the dimeric hepatitis C peptidase 2 or NS2/3 (CPro-1, C18.001), which exhibits a unique fold with two composite His-Glu/-Cys catalytic triads [274]. In addition to inhibition by tosyl-phenylalanyl chloromethyl ketone, a very comlex uAA compound was an effective inhibitor, comprising an expoxide core, a carboxyamide and larger aromatic ring systems with an amino group. Due to the covalent, irreversible inhibition, only an EC_50_ value of 96 µM for autoprocessing was measured [275].

### 3.4. Aspartic Proteases

**Figure 7:**
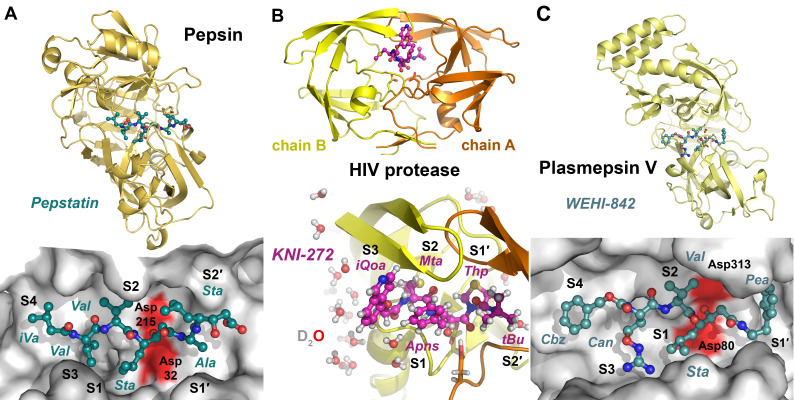
Aspartic proteases in complex with ncAA inhibitors. (**A**) Human pepsin in complex with the bacterial inhibitor pepstatin from *Actinomycetes* (K_i_ = 100 pM), comprising an isovaleryl acetyl cap (P4-iVa) and two statin residues (P1 and P2′ -Sta), as observed in an X-ray structure (PDB 1PSO). The inhibitor is shown as a ball-and-stick model, and the catalytic Asp32 and Asp215 are indicated as red patches in the lower panel. Statin is a β-hydroxy-γ-amino acid with a side chain in the γ-position that corresponds to the one of Leu. (**B**) HIV protease or retropepsin is active as a homodimer with both Asp25 of chain A and B as catalytic residues. Kynostatin (*KNI-272*, ball and sticks) inhibits HIV protease with a K_i_ of 5.5 pM, but it was not effective as a pharmaceutical compound. The tripeptidic core consists of the three ncAAs methylthioalanine, allophenylnorstatine (Apns) and thioproline (Thp), with two capping groups (iQoa and tBu) (PDB 1HPX). A neutron diffraction study showed the positions of hydrogen atoms (white spheres) and of deuterated water molecules (lower panel, PDB 2ZYE). (**C**) The malarial plasmepsin V in complex with the highly potent inhibitor WEHI-842 (ball and sticks). This inhibitor occupies the S4 to S2′ pockets with its peptidic core Can-Val-Sta and the two capping groups carboxybenzyl (Cbz) and phenetylamine (Pea) (PDB 4ZL4).

Typical aspartic proteases such as the prototypic pepsin (A01.001) possess a pair of catalytic Asp residues and nearby stabilizing residues such as Ser, Thr and Tyr [276]. Pepsin was discovered in the early 19th century. It was among the first proteins to be crystallized and was the first one with significant X-ray diffraction. Since the mid-1970s, the natural pepsin inhibitor pepstatin from various *Actinomyces* bacteria has been characterized, with the sequence isovaleryl-Val-Val-Sta-Ala-Sta, containing the ncAA statin, which is essentially a Leu with a CH_2_-OH insertion between the Cα and the carbonyl C [277]. A complex structure of human pepsin with pepstatin was determined by X-ray crystallography by the group of Michael James (Figure 7A) [278]. The synthetic derivative Ac-Val-Sta-Ala-Sta inhibited porcine pepsin with a K_i_ of 1 nM, which is close to that of pepstatin at about 100 pM. Beta secretase 1 (BACE1, A01.004) is a membrane-anchored pepsin-like aspartic protease, which prefers hydrophobic recognition stretches and cleaves the amyloid precursor protein (APP) as a critical step in the development of Alzheimer’s disease. In the search for optimal substrates for in vitro assays, a profiling study found that the dodecapeptide Ile-Ser-Glu-Ile-Thi-Thi↓Nva-Ala-Glu-Phe-Arg-His-NH_2_ was the best substrate, comprising β-2-thienylalanine (Thi) and norvaline (Nva) (Figure 2) [279]. The transmembrane multidomain γ-secretase, with its aspartic protease subunit presenilin-1 (A22.001), was efficiently inhibited by peptidomimetics comprising hydroxyethyl urea side chains at the P1′ and/or P3′ positions, whereas amyloid beta (Aβ) production was significantly reduced [280]. Moreover, inhibition of γ-secretase-dependent Notch signaling promoted the differentiation of neuroblastoma cells, with implications for tumorigenesis and malignancy.

Polypeptidic inhibitors of the aspartic peptidase renin (A01.007) with antihypertensive potential were derived from the hexapeptide pepstatin. Also, its shorter derivative norstatin in the P1 position conferred nanomolar inhibition in compounds with P3 1-Nal [281]. Renin inhibitors, derived from angiotensinogen residues 8–13, were synthesized with two uAAs, e.g., Phe(OMe) in the P3 and P2 position, Sta and two pseudo-dipeptides in P1-P1′ and P2′-P3′ [282]. In addition, when incorporated into Boc-Tyr(OMe)-His-AHNA-OEt, a renin inhibitor with β-hydroxy-γ-amino acids, such as 4-amino-3-hydroxynonanoic acid (AHNA), turned out to be a potent compound with an IC_50_ of 13 nM, and the related compound Boc-Tyr(OMe)-MeLeu-AHPPA-ε-Ahx-Iaa had an IC_50_ of about 1 nM [283,284]. Due to drug resistance, new inhibitor strategies are constantly developed against viral proteases involved in diseases like AIDS and hepatitis [285]. Early studies on inhibitors of aspartic HIV protease (retropepsin, A02.001) showed that tetrahydrofuranyl glycine (Thfg) increases the binding capacity compared with P2-Asn of the compound *Ro31–8959* [286]. Based on these previous studies, P2-Thfg and P3-pyrazine amide residues yielded corresponding inhibitors with IC_50_ values of 70 pM [287]. In similar studies, allophenylnorstatine (Apns or 3-amino-2-hydroxy-4-phenylbutyric acid) was used as a lead building block, with aromatic side chain variations mostly at the P3 and P2′ positions of the HIV protease, resulting in a K_i_ of 500 pM and acceptable pharmacokinetics [288]. This study was continued, mentioning the peptide Ser-Phe-Asp-Apns-Pro-Ile-Val-NH_2_ (P4 to P3′) as a potent inhibitor with a K_i_ of 5 nM [289]. Following these design principles and including other uAAs, HIV protease inhibitors with picomolar potency and antiviral activity were obtained [290]. Extensive structural, functional, and physiological studies were performed for kynostatin (*KNI-272*), a rationally designed inhibitor, which inhibits HIV-1 protease with a K_i_ of 5.5 pM [291]. It comprises the three ncAAs methylthioalanine, Apns and thioproline (Thp), as well as the capping groups 5-isoquinolyloxyacetyl (iQoa) and NH-*tert*-butyl (tBu), thereby mimicking the P3 to P2′ residues of a substrate. In combined X-ray and neutron diffraction experiments, high-resolution structures of the HIV-1 protease iQoa-Mta-Apns-Thp-NH-tBu complex were obtained that showed fine details of the hydrogen atom interactions in the active site (Figure 7B) [292]. Kynostatin reached clinical trial phases I/II, but the effect on HIV-infected patients was unsatisfactory [293]. Ahmpatinin iBu (Ac-Val-Val-Sta-Ala-Ahmppa) isolated from a *Streptomyces* strain significantly inhibited HIV-1 protease with an IC_50_ below 2 nM, with Ahmppa having a methyl-Tyr side chain instead of the Leu equivalent in statin [294]. In addition to preventing the critical dimerization of the two HIV protease subunits by simple lipopeptides with palmitic acid, e.g., Pam-Tyr-Glu-Leu-OH, several tetrapeptides, such as Ac-Ile-Ser-(2Nal)-Glu-Leu-OH, inhibited HIV-1 protease in the nanomolar range [295]. Also, replacing the uAA L-counterpart with Ac-*D*-4F-Phe in the P3 position decreased the IC_50_ about 100-fold to 3 nM [296]. Interestingly, HIV protease inhibitors that comprise very large uAAs with fullerene moieties in the side chain can be quite potent, such as Fmoc-Phe(4-aza-C60)-Hyp-Hyp-Lys-OH, with a K_i_ of 76 nM [297].

Beyond academic research, ncAAs play an important role in the field of antiviral agents and their medical applications. Usually, they are incorporated into peptides and peptidomimetics that inhibit HIV and HCV proteases (hepatitis C virus peptidase, C18.001), such as saquinavir, lopinavir, atazanavir, grazoprevir and glecaprevir [298]. Among these ncAAs are (*S*)-decahydroisoquinoline-3-carboxylic acid amide, (*S*)-Piperazine-2-carboxylic acid, Tle, (2*S*,4*R*)-4-Hydroxy-2-pipecolic acid, 3,3-diphenyl-alanine or (*R*)-5,5-Dimethyl-1,3-thiazolidine-4-carboxylic acid. Also, pentapeptides such as iQoa-Mta-Apns-Dmt-Nbu, in which the critical P1 residue is Apns, inhibit HIV protease with a K_i_ of 2.3 pM [299]. The inhibitor Ac-Phg-Tle-Apns-NH-iBu inhibited HTLV-1 protease (human T-cell leukemia virus-1 protease, A0.2012) in the nanomolar range and, similarly, Apns was crucial in the P1 position of several potent peptidomimetic compounds directed against the aspartic proteases of HIV, HTLV-I and malarial plasmepsin II (A01.023) from *Plasmodium falcipare* and *vivax* [300,301]. In case of the related plasmepsin V (A01.075), which is an integral membrane type I protein, the natural Arg derivative cananavine (Can) in P3 and residues like Chg in P2 conferred inhibitory potency in the low nanomolar or even picomolar range. The crystal structure of compound *WEHI-842* in a complex with the soluble protease domains confirmed the canonical binding (Figure 7C) [302]. Canavanine is translationally active and can replace Arg residues in cell-free protein biosynthesis (Figure 1) [303].

### 3.5. Metalloproteases

**Figure 8:**
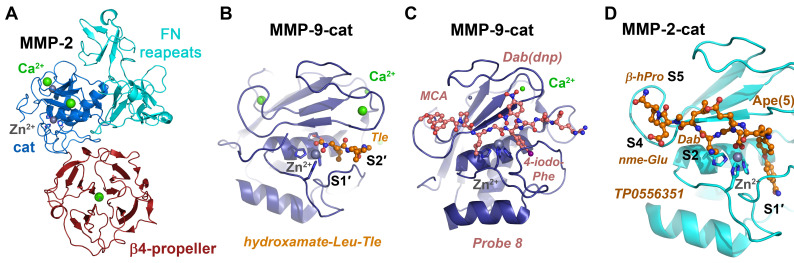
Metalloproteinase (MMP) complexes with ncAA compounds shown as ball-and-stick models. (**A**) Full-length MMP-2 (gelatinase A) comprises a peptidase domain (cat) with the catalytic Zn^2+^ (grey sphere), three fibronectin repeats (FN), a β4-propeller domain and structural Ca^2+^ ions (green spheres), resembling MMP-9 (PDB 1CK7). Like other MMPs, it contains the active site motif HEXXHXXXXXH. (**B**) The catalytic domain of MMP-9 (gelatinase B) with a reverse hydroxamate inhibitor. Leu and *tert*-leucine (Tle) occupy the S1′ and S2′ subsites, respectively (PDB 1GKC). (**C**) MMP-9-cat bound to the fluorogenic substrate *Probe 8* with the sequence MCA-Pro-Leu-Gly-4-iodo-Phe-Dab(dnp)-Ala-Arg-NH_2_. Dab is diaminobutyric acid, N-substituted with dnp being 2,4-dinitrophenyl (PDB 4JIJ). (**D**) MMP-2-Cat displayed in complex with the reversed backbone inhibitor *TP0556351*, which binds the S5 to S1′ subsites with the sequence β-hPro-nme-Glu-Asp-Dab-Glu-Asp-Ape(5)-NH_2_. The β-homo-Pro carries a carboxyamide, nme-Glu is N-methylated, and the aryloxyphenyl group Ape(5) is connected to the Zn^2+^-binding Asp by a (CH_2_)_4_-linker (PDB 7XJO).

Matrix metalloproteinases (MMPs, family M10) constitute a large and important group of Zn^2+^-dependent enzymes that remodel and degrade the extracellular matrix (ECM), in particular various collagens, with additional functions in cell signaling and cancer [304]. They are regulated by the polypeptidic TIMP inhibitors (family I35), as structurally corroborated by the MMP3-TIMP1 complex, which is an example for using SeMet in structure solution (Figure 3A) [53]. MMPs are favored targets of numerous academic and commercial projects that aimed at the discovery of potent pharmaceutical compounds, comprising biosynthesized compounds and in silico screening of inhibitory compounds with ncAAs for several MMPs [305,306,307]. Peptidic compounds such as HS-(CH_2_- *D*-Leu)-Phe-Ala-NH_2_ efficiently inhibit MMP-1 and MMP-8 (fibroblast collagenase, M10.001 and neutrophil collagenase, M10.002), reaching the low nanomolar range [308]. An early example of screening with ncAAs using a positional scanning synthetic library (PSSCL) was the search for gelatinase A (MMP-2) and B (MMP-9) tetrapeptide inhibitors, e.g., His-ε-Ahx-βAla-His, which were efficient in cell cultures, but the in vitro IC_50_ remained in the micromolar range [309]. Among small-molecule inhibitors of metalloproteinases, hydroxamates with variations of the P1′ residue inhibited gelatinase A (MMP-2), gelatinase B (MMP-9), stromelysin-I (MMP-3) and neutrophil collagenase (MMP-8) in the picomolar range, whereby in the P1′ position the following ncAAs conferred high potency: 3-Bal, 1-Nal, 2-Nal, 8-Qal and Tle (Figure 2) [310]. For inhibitor studies, it is favorable to crystallize complexes with the peptidase domain of MMPs, instead of the full-length multidomain protease, which often comprises propeller domains and additional insertions, such as fibronectin in the case of the gelatinases (Figure 8A). A small reverse hydroxamate inhibitor with a P1′-Leu and P2′-Tle was crystallized with the catalytic domain of gelatinase B/MMP-9 (Figure 8B) [311]. Inhibitor fingerprints of MMPs 2, 3, 7, 8, 9, 13 and 14 were obtained from a library of 1400 peptide hydroxamates by systematic variations with cAAs and ncAAs in the P1′, P2′ and P3′ positions [312]. Using the stop codon suppression method, DOPA (3-hydroxy-Tyr) and HqAla (8-hydroxyquinolin-3-yl-Ala) were incorporated into N-TIMP-2 at positions Y36 and S69, respectively, which increased the specific inhibition around 1 nM towards the catalytic domains of MMP-2 and MMP-9, but not for MMP-14 [313]. Highly specific and efficient substrates with the fluorescent groups MCA (7-Methoxycoumarin-4-acetic acid) and DAP ((E)-1-(4-chlorophenyl)-3-[4-(dimethyl-amino)-phenyl]prop-2-en-1-one) were synthesized for MMP-9, comprising the sequence MCA-Pro-Leu-(4-iodo)Phe-DAP-Ala-Arg-NH_2_, which occupies the S4 to S4′ subsites (Figure 8C) [314]. Recently, a peptidomimetic inhibitor called *TP0556351* with an IC_50_ of 200 pM was generated for gelatinase A/MMP-2 (Figure 8D) [315]. It exhibits a β-homo-Pro in the P5 position, followed by (N-Me)Glu-Leu-Dab-Glu and Asp as a Zn^2+^-binding group and a (CH_2_)_4_-aryl-oxyphenyl as an S1′ occupying moiety. Very efficient substrates for MMP-11 (stromelysin-3) and MMP-14 (membrane-type 1 MMP) were generated by placing a S-para-methoxybenzyl-Cys (MeOBn-C) in the P1′ position, resulting in nearly 10- and 40-fold higher catalytic efficiency than the P1′-Leu substrates [316]. Several unusual nncAAs were investigated in this study, such as pPhe, an hPhe extended by a CH_2_ group, or amino acids with n-pentane or n-hexane side chains. A mutational study of the S1′ pocket shaping residue Gln215 in MMP-11 showed that the turnover of fluorogenic heptapeptides was increased at least 25-fold when the P1′ residue Leu was replaced by Cys-(OMe)benzyl (MeOBn-C) [317]. In agreement with these approaches, the optimized FRET substrate Ac-GRRRK(Dabcyl)-GGAAN-(MeOBn-C)-RMGG-fluorescein was found to exhibit 25-fold better affinity for MMP-11 compared to previously synthesized substrates [318]. In addition to a similarly high affinity for MMP-14, this FRET substrate easily penetrated cell membranes and showed high fluorescence signals in cancer cell lines. Based on previously solved complex structures of MMP-13 (M10.013) with peptides and TIMP-inhibitors, a molecular modeling approach led to the synthesis of “TIMP peptidomimetics” [319]. The most potent one inhibited MMP-13, with an IC_50_ of 21 nM, and consisted of three uAAs: an N-terminal azido-bearing residue derived from Lys, a large residue that had a F_3_C-O-Biphenyl-CH_2_-side chain and an ether derived from Ser with a propionyl group, followed by Ser-Ala. The azide and triple bond allowed to cyclize according to the Cu^+^-catalyzed 1,3-dipolar cycloaddition. In a substrate and inhibitor design study, TACE (TNF-α–converting enzyme or ADAM17, M12.217) preferred Phg and hPhe at the P1′ position, as well as basic AAs at the P2′ position, including Cit and methionine sulfoxide Met(o) [320]. According to these substrate preferences, reverse hydroxamate TACE inhibitors with phenethyl and 5-methyl-thiophene-methyl side chains at P1′ or nitro-Arg at P2′ were generated. Even the stereochemistry of a single P1′ residue has a marked effect on inhibitory potency, as demonstrated for a phosphonamidodithionate with a Glu in the R configuration targeting the Zn^2+^-dependent glutamate carboxypeptidase G from *Pseudomonas* [321].

**Figure 9:**
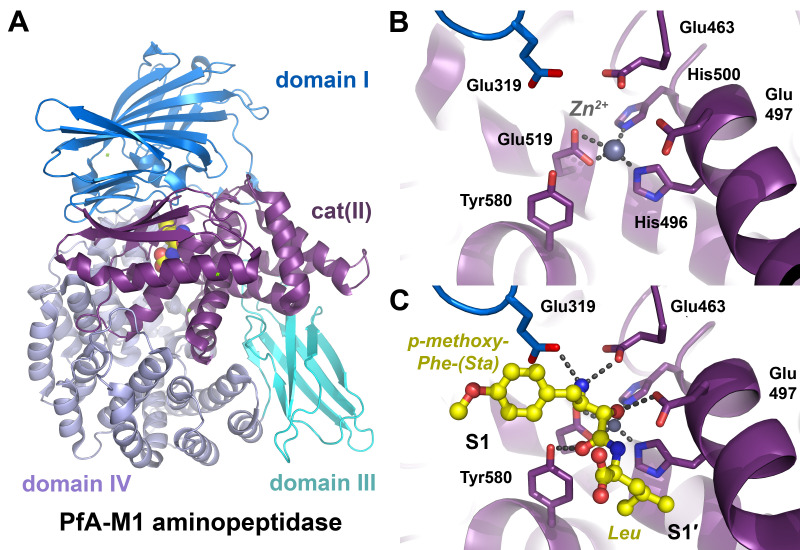
Malarial aminopeptidase PfA-M1 in complex with a bestatin-derived inhibitor. (**A**) PfA-M1 comprises four domains, whereby the inhibitor (yellow sphere model) is located in the center of the catalytic domain cat(II). (**B**) The catalytic Zn^2+^ (grey sphere) is coordinated by His496, His500 and Glu519. Glu497 activates the catalytic water molecule and belongs to the metalloprotease motif HEXXH, which is also present in MMPs, whereas the other side chains bind substrates. (**C**) The inhibitor consists of a modified statin (Sta) with a para-methoxy-Phe side chain instead of a Leu. Its amino group is bound by Glu319 and Glu463 as well as by Tyr580. Though the Sta derivative binds the S1 pocket, Leu is preferred in the S1′ subsite.

Aminopeptidase N or alanine aminopeptidase (APN, M01.001) is a membrane-anchored Zn^2+^ metalloproteinase, which preferentially cleaves P1-Ala and exhibits an unusual specificity for P1′-Trp/Phe [322]. Substrate profiling with kinetic analysis and inhibitor assays showed that the most efficient H_2_N-X-ACC substrates and H_2_N-X-PO_3_H_2_ inhibitors had Nle and hCha P1 residues for both porcine and human APN [323]. APN is regulated by the extracellular adapter protein 14-3-3 binding, whereby phosphorylated peptides, such as APN 36–73 with pSer43 and pThr63, could mimic this specific interaction, which facilitated crystallographic structure determination [324]. The bacterial counterpart from *Neisseria meningitis* (NmAPN) is a potential drug target and was thus analyzed for its specificity in the S1 and S1′ binding sites, including uAAs [325]. The best P1 residue was hArg, and the phosphinate inhibitor hPhe-P-(CH_2_)Tyr exhibited a K_i_ of 54 nM. Similarly, oat (*Avena sativa*) aminopeptidase accepted basic and aromatic residues in P1-ACC substrates, whereby hCha, hTyr, hPhe and hArg were preferred over the best cAA Arg [326]. Bestatin is a natural metalloproteinase inhibitor consisting of an α-hydroxy-β-amino acid with a phenyl ring in P1 and Leu as a P1′ residue, whose potency was increased five-fold by a Tyr(OMe) in P1 with a K_i_ of 43 nM for the PfA-M1 protease (M01.029) of *Plasmodium falciparum* (Figure 9A–C) [327]. A study of the malaria-related amino-peptidases PfM1AAP and PfM17LAP revealed a similar substrate specificity, with the M17 proteases preferring bulky hydrophobic side chains, such as hCha, hPhe and Igl (2-indanyl-glycine) [328]. Methionyl aminopeptidases (MetAP) belong to the M24 family, of which human MetAP1 and MetAP2 as well as *E. coli* MetAP1 were investigated [329]. All three metAPs exhibited an exceptional specificity for Met, which matched hLeu and Nle in the case of both MetAP1s, while corresponding phosphonates inhibited in the low micromolar range. A comparative study on the APN activity of rat, pig and human kidney cell lysates with P1-ACC substrates from HyCoSuL substrates with variations in the P1 position identified hAPN, ERAP1/2 (endoplasmic reticulum aminopeptidases 1 and 2, M01.018/24), IRAP (insulin-regulated aminopeptidase, M01.011), MetAP1 (M24.017) and LTA4H (leukotriene A4 hydrolase, M01.004) [330]. Since ERAP1 and ERAP2 generate a fraction of the MHC-I peptides downstream of the proteasome and contribute to autoimmune diseases, cancer, spondylitis and psoriasis, efficient inhibitors are desired as new lead compounds [331]. Several modified dipeptides, such as hPhe-(PO_2_H)-Leu-CONH_2_, inhibit ERAP1/2, with an IC_50_ in the low nM to pM range [332]. One variant appears as γ-amino acid with a phosphinic acid group in the β-position and two side chains, a combination of NO_2_-hPhe (S1) and Phe (S1′).

A series of α-mercapto acyl-dipeptide inhibitors targeted angiotensin-converting enzyme (ACE, preliminarily designated XM02.001 with two catalytic domains M02.001 and M02.004), which is overall rather unspecific, and neprilyisin (NEP, M13.001) [333]. The most potent compounds reached IC_50_ values of 7 nM (ACE) and 1.2 nM, respectively, and were tested in rats. Systematic variation of the organic residues around the core dipeptide CycL-Tyr-OR containing cyclo-leucine yielded a more potent compound than captopril [334]. Compounds with the proline-derived uAAs Phi and Abo, as present in perindopril and perindoprilate, were investigated as inhibitors of ACE [335]. Efficient inhibitors of ACE and dipeptidyl peptidase 3 (DPP3, M49.001) were generated by replacing the individual P1 residues of the cleavage site in the heptapeptide angiotensin with the cyclic ncAA *cis*-3-aminomethylcyclobutane carboxylic acid (ACCA), whereby the cyclobutane served as a bridging moiety in the backbone [336]. These protease-stable angiotensin variants had a significant effect on cancer cell cultures in reducing their proliferation. More than 70 years ago, studies on antimicrobial peptides demonstrated that Cbz-Gly-Xaa-COOH inhibited mammalian pancreatic carboxypeptidase (M14.001) as well, whereby Xaa can be Thi, 1/2-Nal or *p*-tolyl-*DL*-alanine[337,338].

Eukaryotes such as the fungi *Saccharomyces cerevisia* and *Neurospora crassa* possess a Zn^2+^-dependent mitochondrial processing peptidase (MPP, M16.003), whose activity was studied with synthetic substrates containing P2-Arg and ncAAs in this position [339]. In addition, aminoethoxy acetic acid insertion repeats at the non-prime side enhanced the substrate turnover; however, Cit, Nω-nitro-Arg and others were disfavored.

A metalloprotease that should not be confused with PSA (KLK3) is the prostate-specific membrane antigen (PSMA), the multidomain glutamate carboxypeptidase II (GCPII, M28.010). It is anchored in the membrane and exhibits an unusual specificity for P1-Glu↓P1′-Glu scissile bonds. In order to develop a new inhibitor-directed enzyme prodrug therapy (IDEPT), a multifunctional agent was prepared using the strain-promoted alkyne-azide cycloaddition (SPAAC) of azidophenylalanine (AzF) incorporated into the suicide enzyme yCDtriple and the dibenzylcyclooctyne moiety of the PSMA-targeting agent DBCO-PEG_4_-AH_2_-TG97 [340]. This click reaction product efficiently killed PSMA-positive PCA by converting the prodrug 5-fluorocytosine into the cytotoxic 5-fluorouracil.

Botulinum toxin or bontoxilysin from *Clostridium botulinum* is one of the most toxic biomolecules known and comprises a peptidase domain (endopep, bontoxilysin residues 118-450, M27.002) [341]. Derived from the endopep BoNT/C neuronal transmitter substrate SNAP-25, a single exchange of P1-Lys to Orn resulted in a 200-fold better substrate [342]. Correspondingly, incorporation of homoserine (hSer) and ornithine (Orn) instead of Ser and Lys at distinct positions of a substrate increased the sensitivity of a bontoxilysin mass spectrometry assay for clinical samples about 170-fold [343].

## 4. Modified Proteases

### 4.1. Incorporation of ncAAs into Proteases for Structure-Functional Studies

Since it is much more sophisticated to incorporate ncAAs into large polypeptides and proteins than in small molecules, the preparation of corresponding altered proteases is still in its infancy. Employing subtilisin from *Bacillus lentus* as model protein, regioisomers such as iso-His were generated via a S156C mutant, which did not significantly alter the catalytic properties [344]. By contrast, the site-specific incorporation of 3NY and 3-chlorotyrosine (3CY) instead of Phe205 near the catalytic center of the subtilisin-like protease KP-43 (S08,123) increased the activity at pH 7 to some extent [345]. Due to its unique recognition sequence ENLYFQ↓G/S, tobacco etch virus protease (TEV, C04.004) is one of most frequently used enzymes in tailoring recombinant proteins. TEV protease was modified with caged tyrosine analogues utilizing a bioorthogonal Pyl-tRNA synthetase/tRNA pair, which facilitated optical control of proteolytic activity [346]. The caged uAA nitropiperonyltyrosine (NPY) replaced Tyr178 in a mutant that abolished the activity upon reconstitution by UV light treatment. Based on an efficient in vitro translation system employing UAG/*amber* codon suppression, the structural role of Asp-25 and Asp-125 in HIV protease was investigated by introducing *threo*-β-methylaspartate and β,β-dimethylaspartate in a site-specific manner, resulting in a specific activity of 0 to 45%, which was explained by perturbation in the S1 and S1′ pockets [347,348]. Similarly, 7-(hydroxy-coumarin-4-yl)- ethylglycine (Hco) was incorporated in the NS2B–NSB3 protease of West Nile virus by cell-free protein synthesis via *amber* codon suppression. This unnatural variant of the protease allowed for sensitive measurements of fluorescence upon the binding of inhibitors and small ligand molecules (Figure 2) [349]. In addition, the alkaline protease from *Pseudomonas aeruginosa* (aeruginolysin, AprA, M10.056), a metzincin of the metalloendoproteases, was also modified by replacing the highly conserved Met214 with the uAA difluoromethionine (DFM), which had little effect on the structure and function of the protease [350].

The uAA Nε-4-azidobenzyloxycarbonyllysine (PABK) was used for bioorthogonal ligation by CUAAC. PABK is both a photo-affinity reagent and a chemical decaging compound via the strain-promoted alkyne-azide cycloaddition (SPAAC), enabling intracellular protein activation (Figure 2) [351]. This approach was applied to a model organism, namely, zebrafish embryos, expressing the exogenous blotched snakehead virus protease VP4, possessing a catalytic Ser/Lys dyad in which the lysine was replaced by PABK [352]. Upon decaging by 2-(diphenylphosphanyl)-benzamide, the activated protease cleaved a cytosolic substrate in developing zebrafish, resulting in a clear signal in the nucleus.

In a ground-breaking approach, genetically encoded 2,3-diaminopropionic acid (Dap) was incorporated into the model proteases TEV (C04.004), HtrA-2 (S0.278) and intramembrane RHBDL3 (formerly RHBDL4, rhomboid-like 3 protease, S54.006), replacing the catalytic Cys or Ser (Figure 2) [353]. After photoactivation, the proteases captured both known and novel substrates in complex mixtures in vitro and in mammalian cells by forming stable amide bonds instead of the otherwise transient acyl intermediates, permitting their purification and identification.

### 4.2. Cross-Linking with ncAAs

Site-specific cross-linking is a highly desired approach to investigating protein interactions in structural proteomics and in living cells [354,355]. For more in-depth details on this topic, see the highly topical review of Aydin and Conin, since our focus is on examples of proteases [356]. Thus, keratinase KerBL (subtilisin Carlsberg, S08.001) from *Bacillus licheniformis* was internally cross-linked by the reaction of the uAAs bromo-propyl/butyl-Tyr with Cys mutants, which stabilized flexible regions [357]. Besides having a melting temperature that was more than 10 °C higher for the variants N159C/Y260BprY and N159C/Y260BbtY, their keratinolytic activity nearly doubled, and the catalytic efficiency for the substrate suc-AAPF-pNA did not change significantly. A recent study used ncAA mutagenesis in the heavy chain complementarity-determining region 3 (CDR-H3) of antibody fragments directed against human rhinovirus 14 (HRV14) 3C protease (family C30) [358]. Covalent links of Lys, His or Cys residues in the 3C protease were formed mainly with Tyr106 Fab mutants, having tyrosine analogues or aliphatic chains with bromine or iodine.

The photo-crosslinker DiZPK (3-(3-methyl-3H-diazirine-3-yl)-propamino-carbonyl-Nε-Lys), an analog of pyrrolysine (Pyl), was incorporated into the *E. coli* protease DegP (HtrA, S01.273) using the *amber* stop codon suppression method, using a specific DiZPK-tRNA and a DiZPK-tRNA synthetase (Figure 2) [359]. This approach enabled the cross-linking of periplasmatic interaction partners with DegP, a membrane-anchored elastase-like protease that is crucial for *E. coli* to survive acidic stress [360]. Moreover, the oligomerization state of DegP was analyzed by photo-crosslinking based on the uAAs DiZPK- or Bpa, confirming the formation of dimers and trimers in living cells [361]. Similarly, photo-activatable AzF was incorporated at positions 463 and 466 in the transmembrane segment of BACE1, which trimerized in human cell cultures upon UV irradiation, showing the Cu^2+^-sensing ability of this APP cleaving secretase together with fluorescent labels [362].

### 4.3. Click Reactions with Proteases and Their Inhibitors

**Figure 10:**
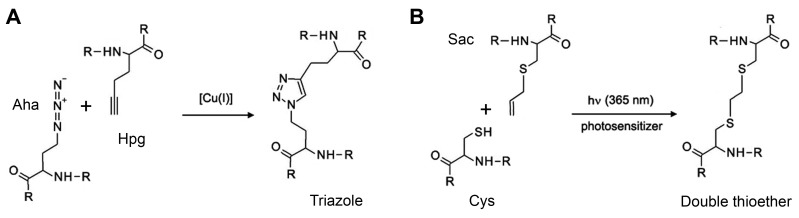
Click reactions that take place under mild conditions in water or even in biological systems. (**A**) In the Cu(I)-catalyzed 1,3-dipolar azide-alkyne cycloaddition, methionine analogs, such as azidohomoalanine (Aha) and homopropargylglycine (Hpg), form stable triazoles by Cu^+^ catalysis in aqueous solution; R represents arbitrary polypeptides. (**B**) The thiol–ene coupling is a photoreaction of a thiol from Cys and an olefin, e.g., S-allylcysteine (Sac), leading to the formation of double thioether links, which are more stable than a disulfide bridge.

The site-specific incorporation of ncAAs into proteins for click reactions with the classical Cu(I) catalyzed click reaction of alkynes and azides (CuAAC) is well established, e.g., for labeling recombinant proteins with biotin or fluorophores (Figure 10A) [363]. Other applications concern additional covalent linkers in polypeptides that increase the stability against proteolytic degradation, as demonstrated for triazole-stapled BCL9 α-helical peptides, which were generated by the classical Cu^+^-catalyzed click reaction of alkynes and an azide (CuAAC) [364]. A specific example of enhanced resistance to the ubiquitin (Ub)-specific protease 1 (USP1, C19.019) was observed for the poly-Ub chains that were generated by the CuAAC reaction of engineered human Ub with Gly76Aha (azidohomoalanine) and propargyl-derivatized Lys27/29 (Plk) [365].

Using double mutants of the Zika virus NS2B-NS3 protease, such as N48AzF/R169AzF, the Gd(III) ion chelator propargyl-DO3A was covalently linked with AzF side chains by the 1,3 dipolar cycloaddition [366,367]. Similar covalent linking via disulfide formation enabled individually labeled constructs to measure double electron–electron resonance (DEER/EPR), which eventually confirmed the closed conformation of the ligand-free and inhibited protease. Furthermore, the cysteine protease inhibitor cystatin B and other proteins in synaptosomes from rat cerebral cortex could incorporate Hpg within two hours, enabling a click reaction with biotin-azide and the subsequent high affinity binding to streptavidin-linked dynabeads for further analysis [368].

As a proof of the principle, it was demonstrated that AzF could be incorporated at basically any position of the yeast multisubunit 26S proteasome, by using the stop codon suppression method and facilitating further click reactions (Figure 2) [369]. Accordingly, reconstituted 26S proteasomes were covalently linked at these distinct AzF positions to dibenzocyclooctyne-linked fluorophores, allowing for the performance of FRET- and anisotropy-based assays for substrate interactions and concomitant conformational changes of the proteasome [370]. In addition, the proteasome-Pup (a ubiquitin equivalent) system of *Actinobacteria* was studied with pupylated proteins conjugated to synthetic peptides and uAAs employing the classical Cu(I)-catalyzed 1,3-dipolar cycloaddition of an alkyne group in Pup and an azide group in the peptide, resulting in the so-called Pup-click [371].

Recently, it was shown that the site-specific incorporation of numerous ncAAs into model proteins can be expanded to a library of aliphatic and particularly small ncAAs with short side chains [89]. Starting with the tRNA^Pyl^ containing the anti-*amber* stop codon and specific PylRS from various archaean organisms, even the relatively short ncAA S-allylcysteine (Sac) has been incorporated, enabling photoclick reactions with Cys-SH groups and many related ncAAs, as well as azido and alkyne group containing ncAAs, such as AzF (Figure 2 and Figure 10B) [372]. Further progress was made by engineering psychrophilic PylRS orthologs from cold dwelling archaea to develop “cold” orthogonal translation systems (OTS) as an alternative to the commonly used meso- and thermophilic OTSs. Apparently, the psychrophilic homolog of PylRS of *Methanococcoides burtonii* has high catalytic efficiency and promiscuity, which allows to incorporate many different ncAAs, with higher yields than previously reported [373].

## 5. Conclusions and Outlook

Regarding the early studies with ncAA containing substrates and inhibitors of the prototypic serine proteases trypsin and chymotrypsin, their focus was on analyses of the mechanisms (see Section 3.1.1). This focus shifted to pharmaceutical applications to some extent, when blood coagulation factors like thrombin or the cancer-related KLKs CatA and CatG were investigated (Section 3.1.2 and Section 3.1.3). In addition, more natural ncAAs were discovered in many cyclic natural small-molecule inhibitors of trypsin-like proteases. Medical and pharmaceutical applications were dominating for the infectious disease-promoting flaviviral serine proteases from Zika, Dengue and West Nile viruses (Section 3.1.4). Other serine proteases were not studied in detail, while the only relevant threonine proteases in the proteasome were intensively investigated with ncAA substrates, inhibitors and ABPs, due to their role in the immune system and in cancer (Section 3.2).

Among the cysteine proteases, the 11 cathepsins are involved in Alzheimer’s disease and various types of cancer, whereas the caspases are most important for apoptotic processes (Section 3.3) They are often tested with the same HyCoSul libraries as their serine protease counterparts, based on similar specificity and mechanisms as in serine proteases. Nowadays, the top target is the SARS-CoV-2 MPro, as its inhibition seems most relevant in times of the Corona pandemic. After ncAA-based inhibitor studies of the prototypic pepsin, especially comparable inhibitors of the HIV protease were very successful with respect to clinical trials and pharmaceutical drug production (Section 3.4). It should be mentioned that ncAA-based HIV inhibitors are often highly effective against hepatitis C protease and the HTLV protease, whereas compounds directed against the malaria-related plasmepsins have not reached their full potential. The human MMPs are major targets of small inhibitors with ncAAs, due to their roles in cancer, also the ADAM, ACE and TACE proteases are still interesting targets (Section 3.5).

Currently, modified proteases are quite heterogeneous, but caging and decaging ncAAs are often incorporated into them, which facilitates activation by photo-reactions (Section 4.1). Again, mainly photo-reactive residues are employed for site-specific cross-linking of various proteases with diverse interacting proteins (Section 4.2). Based on the stop codon suppression method, AzF is the preferred residue for the CuAAC click reaction with an alkyne as counterpart and for photo-reactions (Section 4.3). Interestingly, targets from the ubquitin-proteasome are at the forefront of recent research. However, new reaction types, such as the photo-catalyzed thiol–ene reaction, have become available, which offers the advantage that Cys residues can be the reaction partners.

With the development of new methods in the life sciences, new applications for protease research with ncAAs can be found in the literature. For example, in surface-enhanced Raman spectroscopy (SERS) experiments, trypsin activity could be monitored by an increasing signal intensity upon cleavage and solubilization of a Phe and 4-cyano-Phe (CN-F)-containing peptide, which was bound as a monolayer to a gold solubilization surface [374]. Site-specific incorporation of the ncAA acridonylalanine (Acd) in the LexA represssor protease (S24.001) allowed to monitor the interactions in the LexA:RecA complex [375]. Increasingly, ncAAs facilitate the synthesis of protease-activated prodrugs for pharmaceutical applications, for which Poreba provides a comprehensive review, including information on several clinical trials [376].

Future applications of unnatural amino acids in the life sciences seem limitless, especially in the field of proteases. The increased resistance of biomolecules with uAAs to proteolysis gives them an advantage over conventional peptides and proteins. For example, so called α-PNAs, which contain nucleobase amino acid residues with thymine or cytosine attached to the side chain, are very stable in human serum and offer new pharmacological antisense strategies [377]. Information from peptide-based drug discovery studies of caspases, MMPs and granzymes combining natural and unnatural amino acid substrates enables the prediction of protease-specific cleavage sites, as by the PROSPERous web server (https://prosperous.erc.monash.edu/, accessed on 19 August 2023), facilitating and accelerating future protease studies [378,379,380]. A relatively new and expanding area of research is the uAA-dependent optical control of nucleic acid processing, cell signaling, protein–protein interactions and enzymatic activity [381]. Very promising areas for applications of uAAs are medical and pharmaceutical research, especially the domain of vaccine design [382,383,384].

However, the nearly thirty year old proposal of Sidney Benner that the genetic code should be expanded by adding new, unnatural codons has not been consequently investigated, or it has at least not reached the level of successful protein expression, but corresponding approaches will certainly contribute to upcoming explorations [385]. Recent attempts towards a new genetic code with novel bases and 256 quadruplets might open the door for a completely artificial biology including synthetic living cells [109,386]. Nevertheless, the realization of a hypothetical organism operating with such an expanded genetic code faces practical challenges for two primary reasons. First, if the number of encoded amino acids in such a code is too small (e.g., 20 or 30), the code will become so degenerate that mutations (as the driving force of evolution) will become impossible. On the other hand, an excessively large repertoire, for instance around 100 amino acids, could impose such substantial metabolic burdens that the viability of such an “alien organism” is brought into question. Therefore, the use of quadruplets will most likely be limited to the elucidation of academic issues. Nature has probably chosen a code with triplets to create a balance between these two extreme scenarios [387].

However, within the context of triplet codes, significant potential exists for integrating new amino acids into the genetic code. In 2007, as demonstrated by Szostak and collaborators, in vitro ribosome-mediated translation showcased the insertion of 50 diverse non-canonical amino acids into peptide sequences through the native translation apparatus [388]. This strategy enables approximately 70% of the codons to be reassigned, capitalizing on the substrate tolerance of aminoacyl-tRNA synthetases, a feature that underscores the adaptive promiscuity of these enzymes [389,390]. Dieter Soll and coworkers estimated that 30 to 40 sense codons are sufficient to encode the genetic information of an organism, and a large number of sense codons (>20) may be available for recoding with non-canonical amino acids [391].

The expansion of the genetic code with synthetic nucleotides, nucleobases and nucleic acid backbones is another advanced area of research within synthetic biology that aims to expand the existing genetic code by incorporating new types of nucleic acids into DNA and RNA molecules. This expansion goes beyond the four naturally occurring nucleotides (adenine, cytosine, guanine and thymine/uracil) and involves the addition of chemically modified or fully synthetic nucleotides to the genetic code [392].

The future trajectory of this field lies in the integration of orthogonal translation with synthetic metabolism [393]. This approach seeks to minimize the costly external addition of non-canonical amino acids (or nucleobases) by engineering a synthetic metabolism that is compatible with these unique unnatural substrates. Technically, reprogrammed and orthogonalized translation is mainly encoded by translation components on plasmids. The next step is to use genome-editing methods to genomically integrate all components of reprogrammed translation with noncanonical amino acids. The use of these ncAAs or UAAs can be further “fortified” by getting cells “addicted” to them through adaptive laboratory evolution, as recently shown for fluorinated tryptophans and thienopyrrole [394,395,396]. This comprehensive strategy envisions the emergence of synthetic cells or “orthogonal life,” providing promising platforms for novel protease activities and corresponding inhibitors. Similarly, synthetic cells may promote the generation of ncAA containing biomolecules that confer distinct new functions as molecular switches, catalysts, nanoprobes, metabolic regulators or carriers of activatable drugs to distinct tissues, which all seems immensely attractive. Ultimately, this holistic approach has the potential to usher in a new era of synthetic biology characterized by engineered life forms and functional biomolecules [397].

## Appendix A

**Table A1:** IUPAC names and CAS or Nikkaji numbers (*) of natural non-canonical amino acids.

AA	IUPAC Name	CAS Number
Sec	(2*R*)-2-amino-3-selanylpropanoic acid	10236-58-5
Pyl	*N*^6^-[[(2*R*,3*R*)-3-methyl-3,4-dihydro-2*H*-pyrrol-2-yl]carbonyl]-L-lysine	448235-52-7
Hyp	(2*S*,3*S*)-3-hydroxypyrrolidine-2-carboxylic acid (2*S*,4*R*)-4-hydroxypyrrolidine-2-carboxylic acid	4298-08-2 51-35-4
pSer	(2*S*)-2-amino-3-phosphonooxypropanoic acid	407-41-0
pThr	(2*S*,3*R*)-2-amino-3-phosphonooxybutanoic acid	27530-80-9
Orn	(2*S*)-2,5-diaminopentanoic acid	70-26-8
Cit	(2*S*)-2-amino-5-(carbamoylamino)pentanoic acid	372-75-8
ArgSA	(2*S*)-2-[[N′-[(4*S*)-4-amino-4-carboxybutyl]carbamimidoyl]amino]butanedioic acid	2387-71-5
SAM	(2*S*)-2-amino-4-[[(2*S*,3*S*,4*R*,5*R*)-5-(6-aminopurin-9-yl)-3,4-dihydroxyoxolan-2-yl]methyl-methylsulfonio]butanoate	29908-03-0
SAH	(2*S*)-2-amino-4-[[(2*S*,3*S*,4*R*,5*R*)-5-(6-aminopurin-9-yl)-3,4-dihydroxyoxolan-2-yl]methyl-sulfanyl]butanoate	979-92-0
3SA	(2*R*)-2-amino-3-hydroxy-3-oxopropane-1-sulfinate	1115-65-7
hCys/Hcy	(2*S*)-2-amino-4-sulfanylbutanoic acid	6027-13-0
Tau	2-aminoethanesulfonic acid	107-35-7
Sar	2-(methylamino)acetic acid	107-97-1
2AA	2-aminobenzoic acid	118-92-3
Kyn	4-oxo-1*H*-quinoline-2-carboxylic acid	492-27-3
Aad	(2*S*)-2-aminohexanedioic acid	1118-90-7
hSer	(2*S*)-2-amino-4-hydroxybutanoic acid	672-15-1
β-Ala	3-aminopropanoic acid	107-95-9
*D*-Ser	(2*R*)-2-amino-3-hydroxypropanoic acid	312-84-5
*D*-Asp	(2*R*)-2-aminobutanedioic acid	1783-96-6
GABA	4-aminobutanoic acid	56-12-2
DOPA	(2*S*)-2-amino-3-(3,4-dihydroxyphenyl)propanoic acid	59-92-7
Abu/hAla	(2*S*)-2-aminobutanoic acid	1492-24-6
ADMA	(2*S*)-5-(diaminomethylideneamino)-2-(dimethylamino)pentanoic acid	30315-93-6
Aeg	2-(2-aminoethylamino)acetic acid	24123-14-6
Aib	2-amino-2-methylpropanoic acid	62-57-7
Alg	(2*S*)-2-aminopent-4-enoic acid	16338-48-0
Aze	(2*S*)-azetidine-2-carboxylic acid	2133-34-8
Can	(2*S*)-2-amino-4-(diaminomethylideneamino)oxybutanoic acid	543-38-4
Dab	(2*S*)-2,4-diaminobutanoic acid	1758-80-1
Dha	2-aminoprop-2-enoic acid	1948-56-7
hArg	(2*S*)-2-amino-6-(diaminomethylideneamino)hexanoic acid	156-86-5
Met(o)	(2*S*)-2-amino-4-methylsulfinylbutanoic acid	3226-65-1
Nle/α-Ahx	(2*S*)-2-aminohexanoic acid	327-57-1
Nva/Ape	(2*S*)-2-aminopentanoic acid	6600-40-4
Pip	(2*S*)-piperidine-2-carboxylic acid	3105-95-1
Sem/SeMet	(2*S*)-2-amino-4-methylselanylbutanoic acid	3211-76-5
Tem/TeMet	(2(2*S*)-2-amino-4-methyltellanylbutanoic acid	J663.934H *

## Appendix B

**Table A2:** IUPAC names and CAS or Nikkaji numbers (*) of unnatural non-canonical amino acids.

AA	IUPAC Name	CAS Number
Aca	(2*S*)-2-amino-3-(4-aminocyclohexyl)propanoic acid	J1.610.797B *
ACCA	*cis*-(1*S*,3*S*)-3-(aminomethyl)cyclobutane-1-carboxylic acid	1400744-20-8 (HCl)
ε-Ahx	6-aminohexaonic acid	60-32-2
Ama	(2*S*)-2-amino-3-(4-aminomethylcyclohexyl)propanoic acid	-
Amf	(2*S*)-2-amino-3-[4-(aminomethyl)phenyl]propanoic acid	1991-96-4
Anb	5-amino-2-nitrobenzoic acid	13280-60-9
Cha	(2*S*)-2-amino-3-cyclohexylpropanoic acid	27527-05-5
Chg	(2*S*)-2-amino-2-cyclohexylacetic acid	14328-51-9
Cpa	(2*S*)-2-amino-3-(2-cyanopyridin-4-yl)propanoic acid	169949-53-5
CycL	1-aminocyclopentane-1-carboxylic acid	52-52-8
DfeGly	(2*S*)-2-amino-4,4-difluoro butanoic acid	J786.797B *
DFM	(2*S*)-2-amino-4-(difluoromethylsulfanyl)butanoic acid	J1.943.889I *
DfpGly	(2*S*)-2-amino-4,4-difluoro pentanoic acid	-
Dnp-Lys	(2*S*)-2-amino-6-(2,4-dinitroanilino)hexanoic acid	14401-10-6
4F-Phe	(2*S*)-2-amino-3-(4-fluorophenyl)propanoic acid (^19^F)	51-65-0
5F-Trp	(2*S*)-2-amino-3-(5-fluoro-1*H*-indol-3-yl)propanoic acid (^19^F)	16626-02-1
F(gua)	(2*S*)-2-amino-3-[4-(diaminomethylideneamino)phenyl]propanoic acid	2776-36-5
hCha	(2*S*)-2-amino-4-cyclohexylbutanoic acid	116622-38-9
Igl	(2*S*)-2-amino-2-(2,3-dihydro-1*H*-inden-2-yl)acetic acid	155239-51-3
MfeGly	(2*S*)-2-amino-4-fluorobutanoic acid	J1.434.018A *
MLeu	methyl (2*S*)-2-amino-2,4-dimethylpentanoic acid	90104-02-2
1-Nal	(2*S*)-2-amino-3-naphthalen-1-ylpropanoic acid	55516-54-6
2-Nal	(2*S*)-2-amino-3-naphthalen-2-ylpropanoic acid	6960-34-5
Oic	(2*S*,3a*S*,7a*R*)-2,3,3a,4,5,6,7,7a-octahydro-1*H*-indole-2-carboxylic acid	J532.164F *
Phg	(2*S*)-2-amino-2-phenylacetic acid	2935-35-5
Phi	(2*S*,3a*S*,7a*S*)-2,3,3a,4,5,6,7,7a-octahydro-1*H*-indole-2-carboxylic acid	80875-98-5
Sep	(2*S*)-2-amino-3-(4*H*-selenopheno[3,2-b]pyrrol-6-yl)propanoic acid (selenolo[3,2-b]pyrrole)	J1.076.917E *
TfeGly	(2*S*)-2-amino-4,4,4-trifluorobutanoic acid	15960-05-1
Thfg	(2*S*)-2-amino-2-(oxolan-3-yl)acetic acid	J1.827.275J *
Thi	(2*S*)-2-amino-3-thiophen-2-ylpropanoic acid/(2*S*)-2-amino-3-thiophen-3-ylpropanoic acid	22574-47-6/3685-51-6
Thp	(4*R*)-1,3-thiazolidine-4-carboxylic acid	34592-47-7
Tic	1,2,3,4-tetrahydroisoquinoline-3-carboxylic acid	67123-97-1
Tle	(2*S*)-2-amino-3,3-dimethylbutanoic acid	20859-02-3
CN-Phe	(2S)-2-amino-3-(4-cyanophenyl)propanoic acid	167479-78-9
3CY	(2*S*)-2-amino-3-(3-chloro-4-hydroxyphenyl)propanoic acid	7423-93-0
3NY	(2*S*)-2-amino-3-(4-hydroxy-3-nitrophenyl)propanoic acid	621-44-3
Bpa	(2*S*)-2-amino-3-(4-benzoylphenyl)propanoic acid	104504-45-2
Dap	(2*S*)-2,3-diaminopropanoic acid	4033-39-0
DiZPK	(2*S*)-2-amino-6-[3-(3-methyldiazirin-3-yl)propylcarbamoylamino]hexanoic acid	1337883-32-5
Hco	(2*R*)-2-amino-4-(7-hydroxy-2-oxochromen-4-yl)butanoic acid	905442-42-4
NPY	(2*S*)-2-amino-3-(4-(1-(6-nitrobenzo[*d*][1,3]dioxol-5-yl)ethoxy)phenyl)propanoic acid	207727-86-4
PABK	(2*S*)-2-amino-6-(4-azido-benzyloxycarbonylamino)hexanoic acid	2084913-49-3
Aha	(2*S*)-2-amino-4-azidobutanoic acid	120042-14-0
AzF	(2*S*)-2-amino-3-(4-azidophenyl)propanoic acid	33173-53-4
Hpg	(2*S*)-2-aminohex-5-ynoic acid	98841-36-2
Plk	(2*S*)-2-amino-6-{[(prop-2-yn-1-yloxy)carbonyl]amino}hexanoic acid	1215204-46-8
Sac	(2*R*)-2-amino-3-prop-2-enylsulfanylpropanoic acid	21593-77-1
